# Preferential Amplification of Pathogenic Sequences

**DOI:** 10.1038/srep11047

**Published:** 2015-06-11

**Authors:** Fang Ge, Jayme Parker, Sang Chul Choi, Mark Layer, Katherine Ross, Bernard Jilly, Jack Chen

**Affiliations:** 1 Department of Biology and Wildlife, Institute of Arctic Biology, University of Alaska Fairbanks, Fairbanks, Alaska, USA; 2 Alaska State Public Health Virology Laboratory, Fairbanks, Alaska, USA; 3 Alaska State Public Health Laboratories, Anchorage, Alaska, USA

## Abstract

The application of next generation sequencing (NGS) technology in the diagnosis of human pathogens is hindered by the fact that pathogenic sequences, especially viral, are often scarce in human clinical specimens. This known disproportion leads to the requirement of subsequent deep sequencing and extensive bioinformatics analysis. Here we report a method we called “Preferential Amplification of Pathogenic Sequences (PATHseq)” that can be used to greatly enrich pathogenic sequences. Using a computer program, we developed 8-, 9-, and 10-mer oligonucleotides called “non-human primers” that do not match the most abundant human transcripts, but instead selectively match transcripts of human pathogens. Instead of using random primers in the construction of cDNA libraries, the PATHseq method recruits these short non-human primers, which in turn, preferentially amplifies non-human, presumably pathogenic sequences. Using this method, we were able to enrich pathogenic sequences up to 200-fold in the final sequencing library. This method does not require prior knowledge of the pathogen or assumption of the infection; therefore, it provides a fast and sequence-independent approach for detection and identification of human viruses and other pathogens. The PATHseq method, coupled with NGS technology, can be broadly used in identification of known human pathogens and discovery of new pathogens.

Next generation sequencing (NGS) technologies^1^,^2^, including 2^nd^ and 3^rd^ generation DNA sequencing platforms, have started a revolution in genomics and provided opportunities for its broad application in many other fields^3^,^4^,^5^, including the diagnosis of human pathogens^6^,^7^,^8^,^9^,^10^. Examples of NGS application in the fields of virology and infectious diseases include: 1) epidemiology investigation of infectious disease outbreaks^11^,^12^; 2) etiologic diagnosis of viral infections using a meta-genomic approach^13^,^14^; 3) discovery of new human viruses^4^; and 4) discovery of other new pathogenic viruses^15^. Detailed reviews offer an introduction to NGS technology applications in virus discovery and clinical/diagnostic virology^7^,^8^,^10^. However, NGS technology is still a research tool, rather than a diagnostic tool, and cannot be used in current infectious disease diagnostic laboratories due to 1) the scarcity of pathogen sequences in human clinical samples; 2) the necessary subsequent requirement of extensive deep sequencing; and 3) the complexity of bioinformatics analysis required in order to identify the pathogenic sequences. For example, the average viral genome in a human clinical sample is about 1-100 per 10 million human genome sequence reads. Many laboratories have developed various strategies, from consensus PCR assays that use degenerate primers to computational subtraction of large sequence data in order to find possible unknown pathogens, with little success. These “search for a needle in a haystack” strategies have proven to be a very difficult task.

To make NGS technology a practical tool for detecting human pathogens, the key is to greatly increase the presence of pathogenic sequences in a clinical sample. To address this challenge, we developed a method we called “Preferential Amplification of Pathogenic Sequences (PATHseq)” which can be used to preferentially amplify non-human sequences in a clinical sample. This method is based on the following facts: 1) active infection is the result of pathogenic gene expression, which produces RNAs, or pathogenic transcripts; 2) only about 3% of the human genome generates transcripts. Among these, the top 1,000 and 2,000 most abundant human transcripts comprise more than 65% and 72% of all human transcripts, respectively^16^; 3) by selectively excluding the amplification of these abundant human transcripts, we can preferentially amplify pathogenic transcripts in human clinical samples; 4) pathogenic transcripts can be further enriched through subtractive hybridization against a reference (normal) human transcription library (human transcriptome). The PATHseq technology, in combination with NGS technology, has the potential to provide comprehensive and unbiased detection of human pathogens responsible for any infectious disease.

## Results

### The most abundant human transcripts

The recent completion of the Encyclopedia of DNA Elements (ENCODE) project^17^ provides a genome-wide “landscape of transcription in human cells” in 14 different cell lines. Although the size of the human genome is huge (containing over 3 billion base pairs (bp)), it encodes only about 20,000 protein-coding genes, accounting for a very small fraction (approximately 2%) of the genome. Based on the publicly available ENCODE database^16^, the total human large transcripts (>200 bp RNAs) in GM12878 (a cell line that contributed most to the ENCODE database) are 161,999. Among these, 86,248 transcripts are reproducible (in a duplicated experiment). These 86,248 transcripts are defined as human transcriptome (Table 1). A recent report found that most protein-coding genes have one major transcript expressed at significantly higher level than others, and in human tissues these major transcripts contribute almost 85 percent to the total mRNA^18^. Given that the average length of human mRNAs is 1.3 kb^19^, the complexity can be reduced by 26.8 times (3,000,000/(86,248 × 1.3)), if we sequence cDNA instead of genomic DNA. This strategy has been successfully used by several laboratories in search of human pathogens^4^,^5^,^20^. However, this strategy is still impractical for diagnostic laboratories, because the number of human transcripts is still too large compared to the relative scarcity of pathogenic transcripts.

In order to solve this problem, we developed an alternative strategy using the most abundant human transcripts (Table 1). Our strategy relies on the removal of the most abundant human transcripts from clinical samples to selectively enrich the pathogenic sequences and further reduce the sequencing complexity. As shown in Table 1, we found the most abundant 1,000 and 2,000 transcripts comprised about 65% and 72% of all human transcripts, respectively, based on ENCODE data^17^.

### Non-human oligonucleotides

We further developed a computer program to look for specific patterns in the human transcriptome database. We used the following steps to find the shortest unmatched k-mers of nucleotides in the human transcriptome. First, we collated a set of the Ensembl transcript names (e.g., ENST00000387347), and retrieved DNA sequences corresponding to the transcript names from the human cDNA sequences of the Ensembl release 73 available at ftp://ftp.ensembl.org/pub/release-73/fasta/homo_sapiens/cdna. Secondly, we searched for the shortest unmatched k-mers in the collated set of DNA sequences^21^,^22^. The computer program counted k-mers for a given k or the size of substring in a DNA sequence. It started from k = 1, and checked if all of the possible k-mers occurred at least once. It stopped when it reached a k value where there was at least one k-mer that was not found as a substring in the set of transcripts. As predicted, we found that human transcript sequences are not randomly distributed. Using this computer program, we were able to generate a set of 88 8-mer oligonucleotides (Table 2) that do not match the sequences of the 2,000 most abundant human transcripts. This set of oligonucleotides is, therefore, named as “non-human oligonucleotides”. In other words, by using this set of oligos as primers in the construction of cDNA library, we can get rid of 72% of human transcripts from clinical samples, greatly increasing the chance of selectively targeting pathogenic sequences. Theoretically, this set of primers has the probability to amplify any sequences larger than 5,958 bp (4^8^ × 8 /88), which should include almost all human pathogens (both viruses and bacteria). To test how likely this set of short oligonucleotides can cover all known human viruses, we performed an *in silico* analysis and found that among 386 of all known human viruses (Supplementary Information S1), this set of 88 8-mer oligonucleotides can cover 327 (85%) of them. The remaining 59 unmatched human viruses are usually small human viruses (Supplementary Information S2), which includes human immunodeficiency virus 1 (HIV-1). To cover all know human pathogenic viruses, we also developed a new list of 81 9-mer oligos that do not match the top 2,000 most abundant human transcripts while cover all known human pathogenic viruses (see below *In silico* experiment).

Using the same computer program, we generated several sets of 7-, 8-, 9-, and 10-mer oligonucleotides that selectively amplify non-human sequences during construction of the cDNA library (Supplemental Information S7-10), which is summarized in Table 3. As shown in Table 3, there are 197 9-mer oligonucleotides that do not match the sequence of 500 bp 3’end of all 86,248 human transcripts. To make this strategy work, we introduced ddNTP and used a mixture of ddNTP with normal dNTP (1% ddATP in dNTP solution) in the construction of cDNA library. ddNTP lacks the OH needed to continue the elongation of the DNA strand. When ddATP is added to the reaction, the elongation of the strand stops once the ddATP is added to the new strand. Using this set of 9-mer oligos, the likelihood to find a match in a random sequence is 4^9^ × 9 /197 = 11,976 bp. Most human pathogens have larger genome sizes than this.

### Preferential Amplification of Pathogenic Sequences (PATHseq)

**Figure 1:**
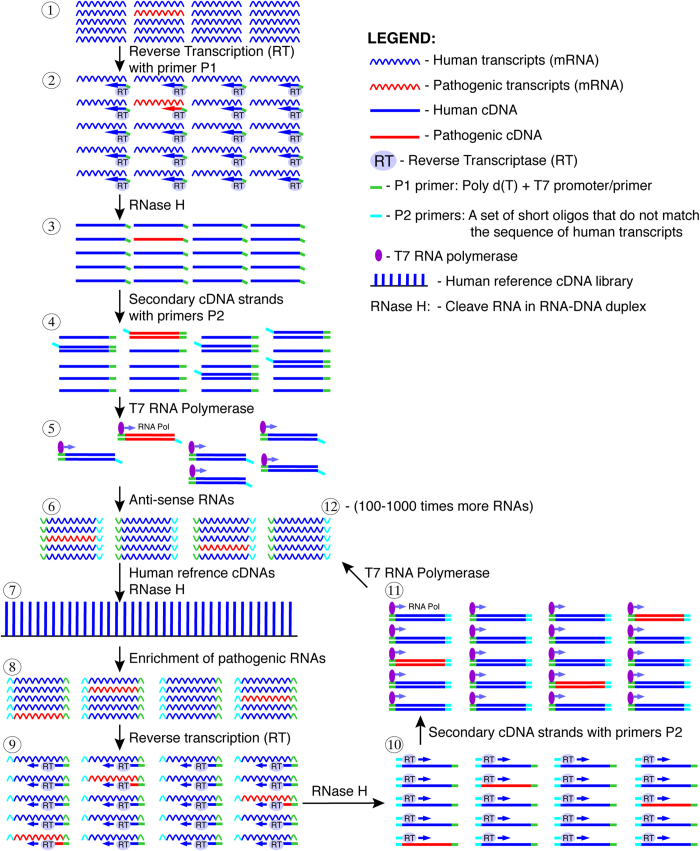
Schematic representation of the PATHseq (Preferential Amplification of Pathogenic Sequences) method. (**1**) Total mRNAs from clinical sample, including human mRNAs and relatively scarce pathogenic mRNAs; (**2**) Total mRNAs are transcribed into first strand cDNAs with P1 primer; (**3**) RNase H cleaves RNAs in RNA-DNA duplex; (**4**) Reverse transcriptase (RT) synthesizes secondary cDNA strands with P2 primers; (**5**) T7 RNA polymerase synthesizes RNAs in the presence of T7 promoter; (**6**) Synthesized anti-sense RNAs; (**7**) Synthesized RNAs are hybridized to human reference (non-pathogenic) cDNA library coated on a solid phase. RNase H cleaves bound RNAs (human RNAs) in RNA-DNA duplex; (**8**) Pathogenic RNAs are enriched; (**9**) Reverse transcription; (**10**) RNase H cleaves RNAs in RNA-DNA duplex; (**11**) T7 RNA polymerase synthesizes RNAs; (**12**) New RNAs synthesized from enriched pathogenic RNAs are amplified 100-1000 fold.

As shown in Fig. 1, PATHseq procedure includes: (1) Total mRNAs are extracted and purified from clinical samples; (2) A primer (P1) is designed to specifically transcribe mRNA into first-strand cDNAs (anti-sense) while introducing a T7 promoter/primer sequence into the cDNA; (3) The ribonuclease activity of RNase H cleaves RNA in a DNA/RNA duplex, allowing the synthesis of secondary cDNA strands; (4) A set of 88 specific 8-mer oligonucleotides (Table 2) is used as primers for the synthesis of secondary cDNA strands. Because these primers do not amplify the 2,000 most abundant human mRNAs, about 72% of all human mRNAs is eliminated from amplification, preferentially amplifying non-human (pathogenic) sequences; (5) Using the T7 promoter introduced in step 2, the T7 RNA polymerase synthesizes RNAs with double-stranded DNA as template; (6) Because the T7 promoter is attached to the poly(A) end, newly generated RNAs are anti-sense; (7) Human reference cDNA library was created using the same set of 8-mer primers (Table 2), plus a poly d(T) primer (not P1 primer). Normal (non-pathogenic) human mRNAs are used as templates when the library is constructed. Sense strands of human reference cDNAs are separated using poly d(T) beads. The beads are further used as the solid phase for subtractive hybridization. Newly generated anti-sense human RNAs from step 6 are captured (hybridized) by these cDNAs and specifically degraded by RNase H in RNA-DNA hybrids. RNase H does not digest single or double-stranded DNA; (8) Pathogenic RNAs are greatly enriched because they do not hybridize to human reference cDNAs; (9) A T7 primer is used to synthesize the first cDNA strands; (10) Again, RNase H cleaves RNAs in a DNA/RNA duplex; (11) Synthesized RNAs are anti-sense; (12) Step 6 through12 form a cycle in which pathogenic RNAs are repeatedly enriched.

### Computational subtraction

**Figure 2:**
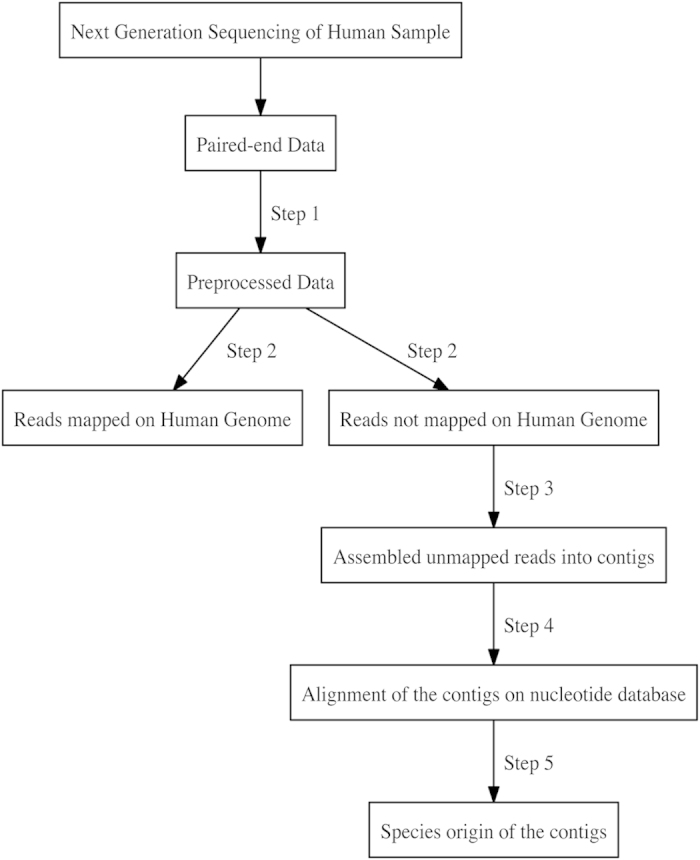
Workflow of identification of sequences with foreign origin. Step 1 is of quality check of the reads, removing low quality reads and trimming low quality bases. Step 2 is to filter out unmapped reads on a host genome. Step 3 is of de novo assembling unmapped reads into contig sequences. Step 4 is to search the BLAST nucleotide sequence database for sequences similar to the contig sequences. Step 5 is to select contig sequences that are mapped on foreign origin sequences in the nucleotide sequence database.

To further analyze sequence data, we developed a computer program to subtract sequencing reads that match human sequences^23^,^24^ and assemble them into contiguous sequences for direct comparison with the GenBank databases of nucleic acids using BLASTN software^25^,^26^. Using this method, any non-matching sequences representing potential pathogenic sequences will be enriched and remain in the final dataset^4^,^5^,^20^. Figure. 2 shows the approach for computational subtraction. In Step 1, to ensure reasonably high quality of the reads, we trimmed the sequences at the 5’-end by 20 base pairs and also trimmed them by quality score from the 3’-end using 30 as the threshold score^27^. In Step 2, using a short read aligner called STAR^28^, we aligned the remaining reads on the human genome of primary assembly sequences unmasked that we fetched from ENSEMBL. Reads aligned to multiple loci in the reference human genome were also considered to be unmapped reads and were filtered out to reduce the false positive rate. In Step 3, to obtain longer non-human origin reads, unaligned reads were assembled using a *de novo* assembler named Trinity^29^. In Step 4, contigs were blasted against NCBI “nr” nucleotide sequence database for sequences that were similar to the *de novo* assembled unaligned sequences^30^,^31^. In Step 5, the assembled unaligned sequences were assigned to species by the taxonomic unit using the NCBI nucleotide sequence database search result and the taxonomy database^32^.

### Proof of concept 1 – enrichment of viral sequences from human cell line

**Figure 3:**
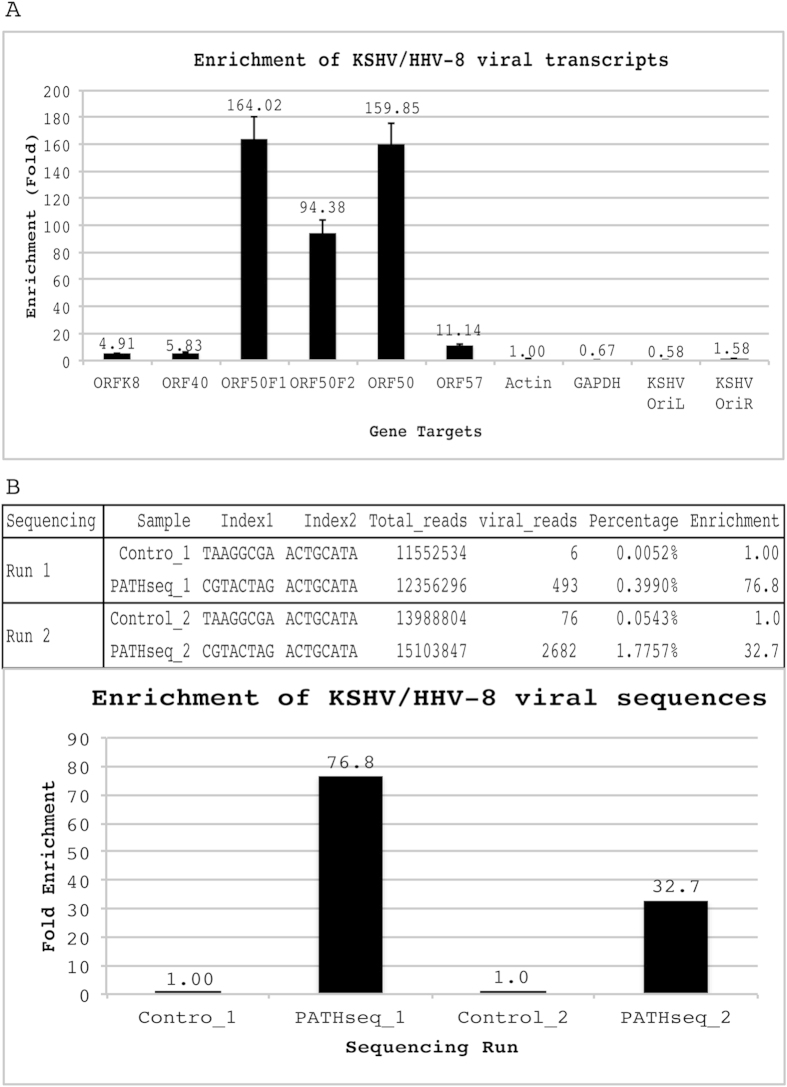
Enrichment of pathogenic sequences by PATHseq method. (**A**) Quantitative real time PCR results indicating enrichment of viral transcripts by PATHseq method from KSHV/HHV-8 latently infected BCBL-1 cell line. ORFK8, ORF40, ORF50F1, ORF50F2, ORF50, and ORF57 are individual lytic viral genes. Actin and GAPDH are cellular house-keeping genes for control; KSHV-OriL and KSHV-OriR are two viral genomic DNA controls; Results are shown from at least three repeats with standard deviation. (**B**) Next generation sequencing results showing enrichments of viral sequencing reads by PATHseq method. Two independent runs using Illumina MiSeq system were performed.

We tested the PATHseq method for the enrichment of pathogenic (viral) sequences from Kaposi’s sarcoma-associated herpesvirus (KSHV) (also known as human herpesvirus 8 or HHV-8)-infected BCBL-1 cells using two different approaches, quantitative real time PCR (qPCR) and next generation sequencing. For qPCR, we designed and optimized KSHV-specific primer sets to monitor the enrichment of viral transcripts compared with cellular house-keeping genes, beta-actin and GAPDH, as controls of background human sequences (Table 4). We also included two viral genomic DNA controls, KSHV-OriL and KSHV-OriR. These two viral genomic locations do not generate viral transcripts. As shown in Fig. 3A, we measured relative enrichment of various viral transcripts from at least three independent experiments compared with the cellular house-keeping gene control, beta-actin. Up to 164-fold enrichment (ORF50F1) could be enriched by PATHseq method. The different enrichment level among various viral genes can be explained as the result of viral transcript size and viral sequence matches with 8-mer oligonucleotides used in PATHseq method.

We further tested the PATHseq method for the enrichment of KSHV viral sequences by next generation sequencing. As shown in Fig. 3B, we performed two independent NGS runs, each consisted of a control (non-enriched) and PATHseq-enriched sequencing libraries. For Run 1, 6 and 493 viral reads could be identified from a total of 11,552,534 and 12,356,296 sequencing reads for control and PATHseq libraries, respectively. For Run 2, 76 and 2,682 viral reads could be identified from a total 13,988,804 and 15,103,847 reads, respectively. The overall enrichments of KSHV viral reads among total sequenced reads increased from 0.0052% to 0.399% (76.8-fold) and 0.0543% to 1.7757% (32.7-fold), respectively.

### Proof of concept 2 – enrichment of viral sequences from virus-spiked human samples

**Figure 4:**
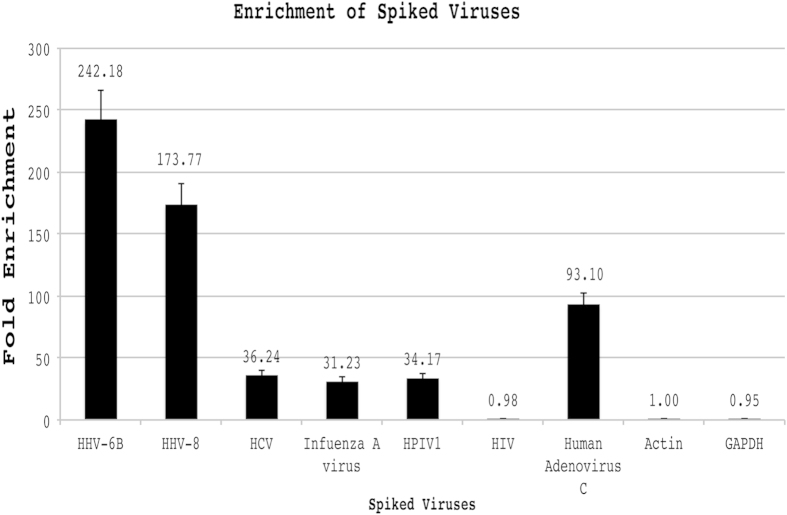
Enrichment of spiked viral sequences. Human mRNAs isolated from peripheral blood mononuclear cells (PBMC) were spiked with viral mRNAs from cell culture infected with human herpesvirus 6B (HHV-6B), human herpesvirus 8 (HHV-8), hepatitis C virus (HCV), Influenza A virus, human Parainfluenza Virus Type 1 (HPIV1), human immunodeficiency virus (HIV), and human Adenovirus Type C. Beta-actin and GAPDH are cellular controls. PATHseq method was performed to enrich the viral sequences. qPCRs were used to monitor the relative enrichments (in fold) of individual virus to cellular gene control, beta-actin. Results are shown from at least three repeats with standard deviation.

We tested the PATHseq method for the enrichment of viral sequences from human samples spiked with multiple viruses. Whole blood was obtained from a healthy blood donor and peripheral blood mononuclear cells (PBMC) were isolated from whole blood using standard procedure. Total RNAs from either PBMC or virus-infected cells were extracted and purified using Qiagen RNeasy Mini Kit. Total RNAs from infected cells were spiked into human PBMC RNAs with a ratio of 1 to 1,000 based on Nanodrop readings. After enrichment by the PATHseq method, quantitative real time PCR (qPCR) was performed with individual viruses, along with two cellular controls, beta-actin and GAPDH. As shown in Fig. 4, we measured relative enrichment of individual viral transcripts from at least three independent experiments compared with cellular house-keeping gene control, beta-actin, which was set to 1. Various human viruses were enriched differently from 31 fold (Influenza A virus) to 242 fold (for Human herpesvirus 6B). The different levels of enrichment for different viruses could be attributed to the fact that the list of 88 8-mer nucleotides used in the PATHseq method matches disproportionately to different viruses. As shown in Table 5, There are 66 8-mer oligonucleotides that match the sequence of human herpesvirus 6B, and 57, 3, 2, 4, and 14 match sequences of human herpesvirus 8, hepatitis C virus, influenza A virus, human parainfluenza virus, and human adenovirus C, respectively. In contrast, there are no oligonucleotides from the list of 88 8-mer oligos that match the genome sequence of human immunodeficiency virus. As a result, there was no enrichment (0.98 fold) of HIV viral sequences from spiked human sample as shown in Fig. 4.

### Clinical diagnosis of unknown infection

**Figure 5:**
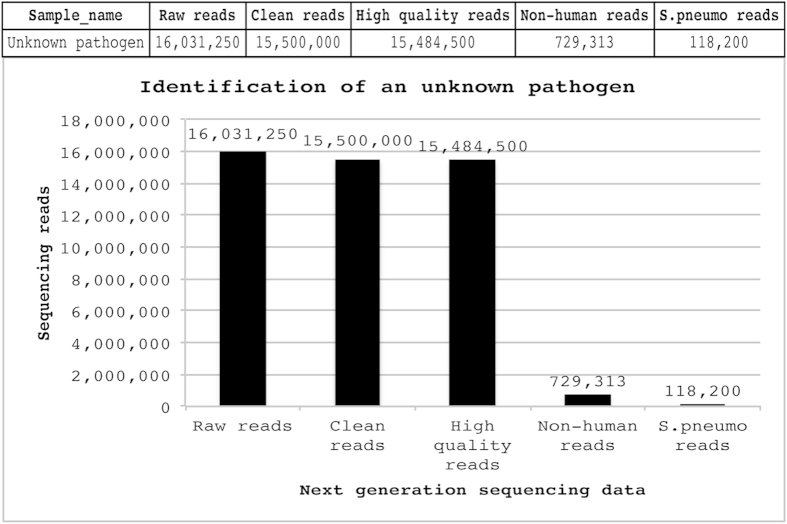
Application of PATHseq method in identifying an unknown infection. Initial raw sequencing reads were 16,031,250, after subtracting human sequences, 118,200 (0.7%) reads were identified as *S. pneumonia* sequences.

The PATHseq method was further tested by investigating an unknown clinical respiratory infection and successfully identified a new variant of *Streptococcus pneumonia* (ASVL_JC_001) from a clinical specimen with bronchitis & pulmonary inflammation^33^. Using PATHseq coupled with NGS, we generated a total of 16,031,250 sequencing reads and eventually identified 118,200 (0.73%) reads as *S. pneumonia* sequences (Fig. 5). These reads formed clusters enriched by the 88 8-mer oligonucleotides and further assembled into 2067 contig sequences. Sequencing analysis of this strain shows atypical features of *S. pneumonia* as it shows alpha-hemolytic colonies (Supplemental Information S3) and bile solubility but was resistant to optochin (Supplemental Information S4). Antimicrobial susceptibility testing shows that this strain was sensitive to cefotaxime, chloramphenicol, oxacillin, penicillin, tetracycline, and vancomycin, but resistant to erythromycin and ethyl hydrocupreine (Taxo P), and partially resistant to sulfamethoxazole trimethoprim (Supplemental Information S4). Metabolic biochemical assay indicated that this variant behaved more like *Streptococcus pneumonia* than to closely related specie *Streptococcus mitis* (Supplemental Information S5). PCR assay of housekeeping genes for *S. pneumonia* indicated that this variant harbors genes encoding the virulence factors pneumolysin (*ply*) and the major autolysin (*lytA*), both of which are normally associated with pneumococci (Supplemental Information S6). Whole genome sequencing was performed on an Illumina MiSeq version 2 system^33^. The total genome of this variant is 2,092,532 base pairs long, and has a GC content of 40.3%. We annotated the genome assembly by using the NCBI Prokaryotic Genomes Automatic Annotation Pipeline^34^. Among a total of 83 contigs, 69 contigs harbor annotations of genes, CDS, and RNAs. We found 2158 putative genes and 2051 CDS. We also found 5 rRNAs, 44 tRNAs and 1 ncRNA for this variant^33^.

### *In silico* experiment

We performed *in silico* experiment to test how likely the PATHseq method can enrich known human viral pathogens. We first generated a set of 199 human pathogenic viruses based on NCBI and ViralZone human viruses databases (viralzone.expasy.org/) (Supplementary Information S11). As summarized in Table 6, we generated the sets of 8-, 9-, and 10-mer oligonucleotides that do not match the most abundant human transcripts at top 1000, 2000, 3000, 4000, 5000, 6000, 7000, 8000, 9000, 10000, 20000, and all 75987, respectively. For example, there are 329 8-mer oligonucleotides that do not match the top 1000 most abundant human transcripts. Among these 329 oligos, there are 62 that match at least one known human pathogenic virus and cover 86.9% of all human pathogenic viruses. Please note there are some minor differences in the total number of human transcripts between Table 5 (75,987) and Table 1 and 3 (86,248) because Table 5 used the UCSC Genome Browser assembly (ID: hg38), while Table 1 and 3 used ENCODE database^16^. Please also note that the total number of human viruses (386) (Supplementary Information S1) is different from the total number of human pathogenic viruses, which is 199 as listed in Supplementary Information S11. Overall, this *in silico* analysis indicates that by recruiting the 81 9-mer oligos (Table 6), we can exclude the top 2000 most abundant human transcripts while still covering 100% of known human pathogenic viruses including HIV, which was missed from coverage by 88 8-mer oligos (Table 5 and Fig. 4). By recruiting the 171 10-mer oligos, we can exclude the top 20,000 most abundant human transcripts while still covering more than 95% of known human pathogenic viruses. Because the rest 55,987 transcripts only count for less than 5% of all human transcripts, none of them would have more abundance than the viral genome.

### Maximal number of sequencing samples

The maximal number of samples that can be loaded onto a sequencing run depends on two factors: 1) the minimal sequencing coverage required for successfully identifying a pathogen, usually the sequencing depth to achieve at least 1 RPKM (Reads per kilobase of transcript per million mapped reads) of pathogen sequences is required; 2) the capacity of the sequencing instrument. Using the same computer program we developed, we generated a total of 40 10-mer oligonucleotides that do not match the sequences to any of the total 161,999 human transcripts (Table 7). These oligos can be divided into two sets and used as adaptors for the construction of sequencing libraries. The maximum combination of these 20 + 20 adaptors can generate 400 (20 × 20 = 400) sample identifiers (barcodes) from A1 to T20. Therefore, a maximum total of 400 samples can be separately labeled by these two sets of adaptors, mixed into one sample run for sequencing and then separated by its own sample identifier (barcode) from A1 to T20 (Table 7).

## Discussion

Next-generation sequencing technology provides broad detection of infectious agents in a sequence independent manner and is rapidly being adapted for the clinical diagnosis of human pathogens. However, NGS technology is still a research tool, rather than a diagnostic tool, and cannot be used in current infectious disease diagnostic laboratories due to the scarcity of pathogen sequences in human clinical samples and necessary subsequent deep sequencing and intensive bioinformatics analysis in order to identify the pathogenic sequences. To solve this problem, many laboratories have developed various strategies. A ribosomal RNA depletion method is wildly used in RNA-sequencing (RNA-Seq) because large ribosomal RNA (rRNA) constitutes approximately 90% RNA species in total RNA^35^,^36^,^37^,^38^. We compared commercial rRNA depletion method (Epicentre’s Ribo-Zero rRNA Removal Core Kit, Cat. No. RZC110424) with our PATHseq method. The rRNA depletion method does not distinguish human RNA from pathogenic RNA, therefore, the amount of human RNA is still overwhelming in the final sequencing library. However, we believe the best way is to combine these two methods together, i.e. to recruit rRNA depletion prior to the application of PATHseq method, instead of polyA enrichment, because some viruses do not produce mRNA. Other methods for enrichment of pathogenic sequences include a capture-based approach using virus-specific DNA fragments as probes through hybridization^39^, and an approach using methyl-CpG binding domain (MBD) to separate methylated host DNA from microbial DNA based on differences in CpG methylation density^40^.

The PATHseq method provides an innovative way to preferentially amplify pathogenic sequences over host sequences through the use of non-human but pathogenic-specific short primers. Theoretically, these primers are short enough to broadly detect pathogens (viruses and bacteria) above a given threshold genome size. For example, the set of 88 8-mer primers has the probability to amplify any sequences larger than 5,958 bp (48 × 8 /88), which should include almost all human pathogens (both viruses and bacteria). However, we noticed that some small viruses including human immunodeficiency virus, could not be enriched by this set of 88 8-mer primers. In addition, some viruses, which do not produce mRNAs, would be missed by the PATHseq method. To address this problem, we further generated a list of 81 9-mer oligos (Table 6). These 9-mer oligos do not match the top 2,000 most abundant human transcripts while covering all known human pathogenic viruses. Using these short non-human primers instead of random primers in the construction of cDNA library, the PATHseq method enables efficient enrichment of pathogenic sequences. With significant enrichment of pathogenic sequences in the final sequencing library, it is possible that more samples can be put into a single run to reduce overall cost and turnaround time. The PATHseq method, in combination with NGS technology, has the potential to provide comprehensive and unbiased (sequence-independent) detection of human known as well as unknown (novel) pathogens.

## Materials and Methods

### BCBL-1 cell line

The body-cavity-based lymphoma cell line, BCBL-1, is latently infected with Kaposi’s sarcoma-associated herpesvirus (KSHV)/human herpesvirus 8 (HHV-8) with average virus copy number ~50 per cell^41^. BCBL-1 is grown in RPMI1640 (HyClone Laboratories, Logan, Utah) supplemented with 10% fetal bovine serum (FBS) at 37 ^o^C in a 5% CO_2_ condition.

### Human specimen with spiked viruses

Single donor human whole blood was purchased from Fisher Scientific (Thermo Fisher Scientific, Waltham, MA, Cat. No. 50-177-224). Peripheral blood mononuclear cells (PBMC) were purified from whole blood using standard procedure^42^. Infecting cells with human herpesvirus 6B was described previously^43^. Hepatitis C patient serum was diagnosed and processed in this lab. Supernatants from infected cell cultures for Influenza A virus (Cat. No. VR-1736), Human Parainfluenza Virus Type 1 (HPIV1)(Cat. No. VR-94), Human Adenovirus Type C (Cat. No. VR-1) were purchased from ATCC. Total RNAs from either PBMC or supernatant of virus-infected cells were extracted and purified using Qiagen RNeasy Mini Kit (Qiagen Inc., Valencia, CA, Cat. No. 74104). mRNAs were purified using NEB Magnetic mRNA Isolation Kit (Cat. No. S1550S). Total RNAs from HIV transfected Jurket cells were described previously^44^. Ratio of spiked viral mRNAs to human PBMC mRNAs were 1 to 1,000 based on Nanodrop reading.

### Clinical specimen with unknown respiratory infection

Sputum from the lower respiratory tract (bronchi and lungs) was obtained from a patient with an unknown clinical bronchitis & pulmonary inflammation at Alaska State Public Health Laboratories. All experiments were performed in accordance with relevant guidelines and regulations and were approved by the Institution Review Board of University of Alaska Fairbanks. Informed consent was obtained from the subject. Total RNAs were extracted and purified using Qiagen RNeasy Mini Kit (Qiagen Inc., Valencia, CA) with some modification. Briefly, sputum was suspended in 500 μl of lysis buffer with 20 μl of proteinase K (20 mg/ml) and incubated for 1 hour at 56 ^o^C. The sample was further extracted with phenol/chloroform several times until there was no white protein layer between two phases. The clean aqueous phase was then processed according to Qiagen protocol. Purified total RNA was then used to prepare a sequencing library using the PATHseq method described below.

### Procedure for coupling magnetic beads with oligonucleotides

Primer P1 was specifically designed to contain T7 promoter sequence, in addition to poly d(T) sequences (P1: 5’-ACGGCCTAATACGACTCACTATAGGGTTTTTTTTTTTTTTTTTTVN-3’). When synthesized, a primary amino group was attached to the 5’-end of P1 with a standard (C6) spacer arm (Integrated DNA Technologies, Coralville, IA). Modified primer P1 was manually coupled to Pierce NHS-activated magnetic beads (Thermo Fisher Scientific Inc., Rockford, IL), according to the manufacturer’s instruction. The final concentration of P1-coupled magnetic beads was 10 mg/ml.

### Poly(A) mRNA isolation and first strand cDNA synthesis with P1-Magnetic Beads

Total RNA was extracted using Qiagen’s RNase Mini Kit (Qiagen, Valencia, CA) according to the manufacturer’s instruction. 5 μg of total RNA was diluted with nuclease-free water to a final volume of 50 μl in a nuclease-free 0.2 ml PCR tube. 10 μl of P1-Magnetic Beads were washed twice with 100 μl of RNA Binding Buffer (1 M LiCl, 40 mM Tris HCl, pH 7.5, 2 mM EDTA, and 0.1% Triton X-100). Beads were re-suspended in 50 μl of RNA Binding Buffer and added to the 50 μl of total RNA sample. Tubes were placed on the thermal cycler and heated at 65 °C for 5 minutes and held at 4 °C to denature the RNA and facilitate binding of the poly(A) mRNA to the beads. Tubes were removed from the thermal cycler and incubated at room temperature for 5 minutes to allow the RNA to bind to the beads. Tubes were then placed on the magnetic rack at room temperature for 2 minutes to separate the poly(A) mRNA bound to the beads from the solution, supernatant was removed and discarded. Beads were washed twice with 100 μl of Wash Buffer (150 mM LiCl, 20 mM Tris HCl, pH 7.5, 1.0 mM EDTA, and 0.01% Triton X-100) to remove unbound RNA, each time with all the supernatant being removed and discarded. Beads were equilibrated with 100 μl 1x reverse transcriptase (RT) buffer and separated by magnetic field. Beads bound with poly(A) mRNA were re-suspended in 12 μl nuclease-free water, 4 μl dNTP mix (10 mM each), 2 μl of 10x RT buffer, 1 μl of RNAse inhibitor, and 1 μl of M-MuLV reverse transcriptase were added to the solution. Mixture was incubated at 42°C for one hour and then inactivated at 90 °C for 3 min. Magnetic beads with first strand cDNA synthesis were separated and the supernatant being removed and discarded.

### PATHseq

88 octamer (Table 2) and 197 nonamer (Extended Data Table 4) oligonucleotides were synthesized using Fisher Custom Oligos service (Thermo Fisher Scientific, Waltham, MA). Oligos were suspended in nuclease-free water at final concentration of 10 μM. The set of 88 octamer oligos were used in all experiments in this report. Normal peripheral blood mononuclear cells (PBMCs) were isolated from human whole blood of a single male donor (Thermo Fisher Scientific, Waltham, MA). Total reference RNAs were extracted using Qiagen RNeasy Kit. mRNAs were isolated from total RNAs using NEBNext Poly(A) mRNA Magnetic Isolation Module according to manufacturer’s protocols (NEB). Reference cDNA library was constructed using NEB First Strand Synthesis Protocol with M-MuLV Reverse Transcriptase^42^. cDNAs were purified using Qiagen MinElute Reaction Cleanup Kit. To perform PATHseq method, magnetic beads with first strand cDNA synthesis were equilibrated with 100 μl 1x PATHseq buffer (40 mM Tris-HCl, pH 7.9, 20 mM MgCl_2_, 10 mM DTT, and 2 mM spermidine) and separated by magnetic field. Supernatant was removed and discarded. Beads were re-suspended in 8.4 μl nuclease-free water, then the following solutions were added: 2 μl of 10x PATH buffer, 2 μl Octamers (10 μM), 1 μl dNTP mix (25 mM), 3.2 μl rNTP mix (25 mM), 0.4 μl Pyrophosphase (Inorganic (*E. coli*), 100 units/ml, New England BioLabs, Ipswich, MA), 1 μl reference human cDNAs (0.1 μg/1 μl), 1 μl M-MuLV Reverse Transcriptase (NEB), and 1 μl T7 RNA polymerase (NEB). Final volume was 20 μl. Mixture were incubated at 40 °C for 3 hours with gentle agitation. PATHseq cDNA library was purified using Qiagen MinElute Reaction Cleanup Kit.

### Next generation sequencing

Next generation sequencing was performed with Illumina MiSeq Desktop Sequencer version 2 (Illumina, San Diego, CA). Sequencing library was prepared using Nextera DNA Sample Preparation Kit (Illumina) according to the product’s guide. Sequencing run was carried out using Illumina MiSeq Reagent Kit v2 (500-cylces), which generates 24–30 million paired-end reads of 2 × 250 bp length, total output of 7.5-8.5 Gb.

### Sequencing data analysis

Raw sequencing data was filtered by in-house scripts: 1) Remove reads with 3 N; 2) Remove reads contaminated by adapter (default: 15 bases overlapped by reads and adapter); 3) Remove reads with a certain proportion of low quality (20) bases (40% as default, parameter setting at 36 bp); 4) Remove duplication contamination.

Using a computer program called STAR^28^, quality sequencing reads were aligned against the human genome primarily assembled from ENSEMBL (http://uswest.ensembl.org/index.html). Reads aligned to multiple loci in the reference human genome were also considered as unmapped reads and filtered out to reduce the false positive rate. To obtain longer non-human origin reads, unaligned reads were further assembled using a *de novo* assembly computer program named Trinity^29^, resulting in larger contig sequences. The *de novo* assembled unaligned sequences were blasted against the nucleotide sequence database known as NCBI “nr” database. Finally, the assembled sequences were identified using the NCBI genomic BLAST database for “Microbes” including bacteria, fungi, and viruses^32^.

### Quantitative real time PCR (qPCR)

qPCRs were performed with ABI’s StepOnePlus Real-Time PCR Systems. Reactions were set up with ABI’s SYBR Select Master Mix (Life Technologies, Carlsbad, CA) according to product’s instruction.

### Accession numbers

The genome sequence data of *S. pneumonia* strain ASVL_JC_0001 identified in this study has been deposited at DDBJ/EMBL/GenBank under the accession number JJMK01000000, and consists of sequences JJMK01000001 - JJMK01000083.

## Additional Information

**How to cite this article**: Ge, F. *et al.* Preferential Amplification of Pathogenic Sequences. *Sci. Rep.* **5**, 11047; doi: 10.1038/srep11047 (2015).

## Supplementary Material

Supplementary Information

## Figures and Tables

**Table 1:** The most abundant human transcripts.

**Most abundant human transcripts**	**RPKM**	**% of total human transcriptome**
Top 1000	23391.45	65.52%
Top 2000	25847.42	72.40%
Top 3000	27355.52	76.62%
Top 4000	28440.66	79.66%
Top 5000	29287.62	82.04%
Top 6000	29973.64	83.96%
Top 7000	30544.67	85.56%
Top 8000	31035.38	86.93%
Top 9000	31463.82	88.13%
Top 10000	31838.97	89.18%
Top 20000	34018.78	95.29%
All 86248	35700.85	100%

RPKM: Reads per kilobase of transcript per million mapped reads.

**Table 2:** A list of 88 8-mer oligonucleotides that do not match the sequences of the 2,000 most abundant human transcripts.

AAACGCGA	ACGAACCG	ATGCGATA	CCGTAGTA	CGCAATAT	CGGGTCGA	CTAATACG	GTTACGCG	TAGCGAAT	TATCCGAC	TCGTCGAT
AACGCATA	ACGAATAA	ATGCGTTA	CGAACGTA	CGCGATAC	CGGTAAGC	CTTAGCGA	TAACCGTT	TAGCGTAC	TATCGCTA	TGTAAGCG
AATAACGC	ACGATAGG	ATTAGCGT	CGAATAAC	CGCGCGTA	CGGTAGAT	GATACGTA	TAACGTAA	TAGCGTAT	TATCGGAC	TTAACGTA
AATATCGT	ACGCGATA	ATTGCGAC	CGACGTAC	CGCGGTTA	CGGTAGTA	GCGAATAT	TAAGCGCG	TAGTAACG	TATCGGTA	TTACGATA
AATATTCG	ATACCGGT	ATTGTACG	CGATAGGT	CGCGTAAT	CGGTCGAT	GCGACGTA	TAAGGTCG	TAGTCGAG	TATCGGTC	TTAGTCGA
AATCGGTA	ATACGTAC	CAATCGCG	CGATAGTA	CGCGTATA	CGTATATC	GCGTAATT	TAATACGT	TAGTCGGT	TCGAATAG	TTATAGCG
ACACGTTA	ATAGCGCA	CCCTAACG	CGATATCC	CGCGTATC	CGTATTCG	GTACCGTA	TAGAGTCG	TATAGCGC	TCGCGTAT	TTATATCG
ACCGGTTA	ATAGCGCG	CCGGTAAT	CGATCGTA	CGCTAAAA	CGTCGAAT	GTATAACG	TAGATCCG	TATCACGC	TCGGTAAC	TTATCGCG

**Table 3:** Numbers of oligonucleotides that do not match the sequences of human transcripts.

	**Top 1000 transcripts**			**Top 2000 transcripts**			**Top 4000 transcripts**			**All 86,248 transcripts**		
	**Full transcript**	**1000bp -3'End**	**500bp -3'End**	**Full transcript**	**1000bp -3'End**	**500bp -3'End**	**Full transcript**	**1000bp -3'End**	**500bp -3'End**	**Full transcript**	**1000bp -3'End**	**500bp -3'End**
7-mer	1	2	65			4			1			
8-mer				88	455		1	74				
9-mer										1	20	197

**Table 4:** Primers for HHV-8/KSHV quantitative (real time) PCR.

**Name**	**Forward**	**Sequence**	**Reverse**	**Sequence**	**Start**	**Finish**	**gDNA(bp)**	**cDNA(bp)**
ORFK8	K8S-F	AGACAGCTGCAGCAGGCATT	K8-Rnew	CTGCTGGCACATTCGCATCA	75648	75851	203	122
ORF40	ORF40S-F	GCTTTGGAGCCTGAGCAATG	ORF40-R	ATGCGATGAGAATACAAGAT	61742	61982	240	114
ORF50F1	ORF50-F1	AGGTGTGCCGTGTAGAGATT	ORF50-R1	TGCTTTCGTTTGGGTGTTGT	74140	74208	68	68
ORF50F2	ORF50-F2	CGCGCTGTTGTCCAGTATTC	ORF50-R2	CCACCAGAAGGTGACGGTAT	74491	74613	122	122
ORF50	ORF50-F	GCGCAAGATGACAAGGGTAA	RTApath-R2	CAAGCTTGGAACATTCTTTC	71698	74613	2915	199
ORF57	ORF57path-F	AGGGCATCCTAGAGGACTCT	ORF57-R	GGGTTCGGACAATTGCTCGT	82203	82411	208	101
β-actin	b-actin-F	ACGTGGACATCCGCAAAGAC	b-actin-R	CAAGAAAGGGTGTAACGCAACTA	945	1252		308
GAPDH	GAPDH-F	GAAGGTGAAGGTCGGAGTC	GAPDH-R	GAAGATGGTGATGGGATTTC	180	405		226
KSHV-OriL	KSHV-OriL-F	GCTAGTGAGTCACGGGCCTG	KSHV-OriL-R	GTAACAGTTGGTTAACCCGT	23946	24117	171	
KSHV-OriR	KSHV-OriR-F	ATCCGGCCGTCCTGGGCAGC	KSHV-OriR-R	GGGACGAGGAAAAAGTACGC	122167	122228	61	

**Table 5:**
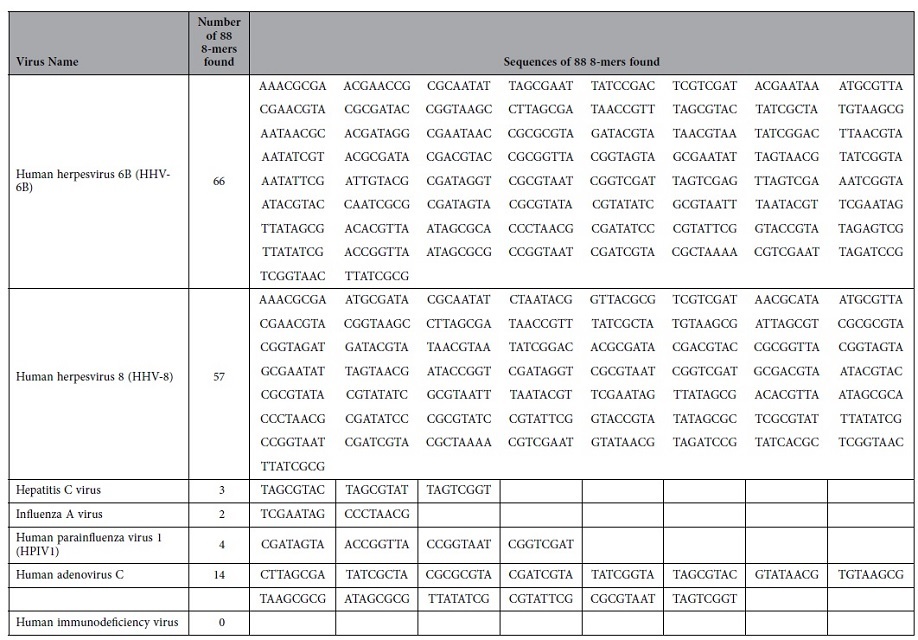
Match of 88 8-mer oligonucleotides in spiked virus genomes.

**Table 6:** In Silico Analysis of human virome coverage by short non-human primers.

**The Most Abundant Human Transscripts**												
	**Top 1000**	**Top 2000**	**Top 3000**	**Top 4000**	**Top 5000**	**Top 6000**	**Top 7000**	**Top 8000**	**Top 9000**	**Top 10000**	**Top 20000**	**All 75987**
Number of 8-mers unmatched to the most abundant human transcripts	329	44	9	4	2	1	1	0	0	0	0	0
Among above 8-mers, number that match at least one known human virus	62	26	8	4	2	1	1	0	0	0	0	0
human virome coverage by above 8-mers	86.9%	41.2%	16.6%	11.6%	9.0%	8.0%	8.0%	0.0%	0.0%	0.0%	0.0%	0.0%
Number of 9-mers unmatched to the most abundant human transcripts	23473	8883	4402	2411	1493	953	651	455	347	249	28	1
Among above 9-mers, number that match at least one known human virus	57	81	87	91	94	85	83	69	62	51	14	1
human virome coverage by above 9-mers	100%	100%	98.5%	94.0%	87.9%	76.9%	70.4%	61.8%	57.8%	46.7%	16.1%	2.0%
Number of 10-mers unmatched to the most abundant human transcripts	351888	203816	139254	100542	76937	60510	49753	41374	35737	30336	10053	1075
Among above 10-mers, number that match at least one known human virus	164	167	174	179	177	180	180	181	179	179	171	81
human virome coverage by above 10-mers	100%	100%	100%	100%	100%	100%	100%	100%	100%	100%	95.5%	48.2%

**Table 7:** Index/Adaptor sequences for sequencing library.

**Adaptor**	**Sequence**	**Sequence**	**Adaptor**		
A	ACGCGTATGA	CGTAATACGT	1		
B	ACGTAGCGTG	CGTAATCGGT	2		
C	ATACGCGACT	CGTACAAACG	3		
D	ATCGACGCAA	CGTACGAAAC	4		
E	ATCGTTCGAC	CGTACGTTAG	5		
F	ATTCGATCGC	GCGCGATAGG	6		
G	CCGTCGAAGT	GCGCGTAAAT	7		
H	CGAACGAATC	GTACGCGACT	8		
I	CGACGTATTG	GTCGAACGAG	9		
J	CGATACGTTC	TAACGTATCG	10		
K	CGATCTAACA	TAACGTCGGC	11		
L	CGATTCGGTT	TACGCGATTG	12		
M	CGCCCGTTAA	TAGCGAACGC	13		
N	CGCGATAGTG	TAGCGACGCA	14		
O	CGCGTGTTAT	TATGCGACGC	15		
P	CGGATCGTTA	TCGATCGGTG	16		
Q	CGGTACGCAT	TCGCGAAATT	17		
R	CGGTCGTAGA	TCGCGAATGA	18		
S	CGTAACGACT	TCGTTCGTAC	19		
T	CGTAACTAGG	TTATCGCGCA	20		
