# Supplementary material for: Preferential Amplification of Pathogenic Sequences
Source: Sci Rep. 2015 Jun 11;5:11047. doi: 10.1038/srep11047 (PMC4464073; doi:10.1038/srep11047)
Supplement: Supplementary Information [file srep11047-s1.pdf]

Supplemental Information - S1-S11

Preferential Amplification of Pathogenic Sequences

Fang Ge[1], Jayme Parker[1,2], Sang Chul Choi[1], Mark Layer[1], Katherine Ross[3], Bernard Jilly[3] & Jack Chen[1,2*]

Author affiliation:

[1] Department of Biology and Wildlife, Institute of Arctic Biology, University of Alaska Fairbanks, Fairbanks, Alaska, USA.

[2] Alaska State Public Health Virology Laboratory, Fairbanks, Alaska, USA

[3] Alaska State Public Health Laboratories, Anchorage, Alaska, USA

Supplemental Information S1: A list of all human viruses, a total of 386.

1. Vaccinia virus
2. Variola virus
3. Human herpesvirus 1
4. Human herpesvirus 2
5. Human herpesvirus 3
6. Human herpesvirus 5
7. Human herpesvirus 7
8. Human herpesvirus 4
9. Hepatitis B virus
10. BK polyomavirus
11. JC polyomavirus
12. Simian virus 40
13. Igbo Ora virus
14. Ross River virus
15. Rubella virus
16. Japanese encephalitis virus
17. Murray Valley encephalitis virus
18. St. Louis encephalitis virus
19. West Nile virus
20. Powassan virus
21. Tick-borne encephalitis virus
22. Langat virus
23. Louping ill virus
24. Yellow fever virus
25. Hepatitis C virus subtype 1b
26. Human coronavirus 229E
27. Mumps virus
28. Newcastle disease virus
29. Sendai virus
30. Human parainfluenza virus 4a
31. Parainfluenza virus 5
32. Human parainfluenza virus 2
33. Bovine parainfluenza virus 3
34. Human parainfluenza virus 3
35. Simian virus 41
36. Canine distemper virus
37. Measles virus strain Edmonston-Zagreb
38. Rinderpest virus
39. Bovine respiratory syncytial virus ATCC51908
40. Human respiratory syncytial virus
41. Murine pneumonia virus
42. Marburg marburgvirus
43. Chandipura virus IB An 9978
44. Vesicular stomatitis Indiana virus
45. Rabies virus
46. Influenza A virus (A/Hong Kong/1073/99(H9N2))
47. Influenza B virus
48. Influenza C virus
49. Influenza C virus (C/Ann Arbor/1/50)
50. Influenza C virus (C/Yamagata/9/88)
51. Thogoto virus
52. Rift Valley fever virus
53. Zaliv Terpenia virus
54. Precarious point virus
55. Chize virus
56. Crimean-Congo hemorrhagic fever virus
57. Dugbe virus
58. Hantaan virus
59. Hantaan virus CA10081206
60. Puumala virus
61. Seoul virus
62. Junin virus
63. Lassa virus
64. Lymphocytic choriomeningitis virus
65. Machupo virus
66. Mopeia virus
67. Pichinde virus
68. Tacaribe virus
69. Human foamy virus
70. Human immunodeficiency virus 1
71. Human immunodeficiency virus 2
72. Norovirus Hu/GI.1/8McIII/1973/USA
73. Borna disease virus
74. Hepatitis E virus
75. Hepatitis delta virus dTk13
76. Dobrava-Belgrade virus
77. Mokola virus
78. Omsk hemorrhagic fever virus
79. Dengue virus 1
80. Human parainfluenza virus 1
81. Respiratory syncytial virus type A
82. Avian paramyxovirus 4
83. Sandfly fever sicilian virus
84. Sandfly Sicilian Turkey virus
85. Rotavirus A
86. Rotavirus A human/Victoria/CK00022/2005/G1P[8]
87. Rotavirus A human/Bethesda/DC130/1976/G3P[8]
88. Human rotavirus G9P[8]
89. Rotavirus A human/Bethesda/CH5477/1991/G3P[8]
90. Human rotavirus A
91. Human rotavirus B
92. Rotavirus B
93. Porcine rotavirus B
94. Peste-des-petits-ruminants virus
95. Cell fusing agent virus
96. Human herpesvirus 6A
97. Human herpesvirus 6B
98. Alkhumra hemorrhagic fever virus
99. Ngari virus
100. Maguari virus
101. Ilesha virus
102. La Crosse virus
103. Chatanga virus
104. Snowshoe hare virus

105. Simbu virus
106. Dolphin morbillivirus
107. Bovine rotavirus C
108. Porcine rotavirus
109. Rotavirus C
110. Human rotavirus C
111. Porcine rotavirus C
112. Bovine group C rotavirus
113. Chikungunya virus
114. Tula virus
115. Human herpesvirus 8
116. Sin Nombre virus
117. Avian metapneumovirus
118. Lagos bat virus
119. Duvenhage virus
120. Oliveros virus
121. Mapuera virus
122. Torres virus
123. Sepik virus
124. Amapari virus
125. Guanarito virus
126. Flexal virus
127. Latino virus
128. Parana virus
129. Tamiami virus
130. Human endogenous retrovirus K113
131. Sabia virus
132. Andes virus
133. Oran virus
134. Maporal virus
135. Colorado tick fever virus
136. Whitewater Arroyo virus
137. Arenavirus H0380005
138. Pirital virus
139. Colobus guereza papillomavirus type 2
140. Porcine rubulavirus
141. GB virus C
142. Ippy virus
143. Mobala virus
144. European bat lyssavirus 1
145. European bat lyssavirus 2
146. Mayaro virus
147. Ilheus virus
148. Eyach virus
149. Hendra virus
150. Apoi virus
151. Entebbe bat virus
152. Rio Bravo virus
153. Usutu virus
154. Karshi virus
155. Bagaza virus
156. Ntaya virus
157. Tembusu virus
158. Yokose virus
159. Modoc virus
160. Aroa virus
161. Kedougou virus
162. Montana myotis leukoencephalitis virus
163. Zika virus
164. Torque teno virus
165. Akabane virus
166. Human erythrovirus V9
167. Banna virus
168. Australian bat lyssavirus
169. Tupaia paramyxovirus
170. TTV-like mini virus
171. Hantavirus Z10
172. Sapovirus Po/OH-JJ681/2000/US
173. Kadipiro virus
174. Human adenovirus 21
175. Oropouche virus
176. Nipah virus
177. Fer-de-lance virus
178. Human adenovirus 31
179. Human adenovirus C
180. Simian adenovirus 22
181. Human adenovirus F
182. Human adenovirus 30
183. Human enterovirus 71
184. Human echovirus 11
185. Human poliovirus 1
186. Human enterovirus 68
187. Allpahuayo virus
188. Human picobirnavirus
189. Human rhinovirus 68
190. Human rhinovirus 84
191. Menangle virus
192. Avian paramyxovirus 6
193. Cat Que virus
194. Ingwavuma virus
195. Douglas virus
196. Aino virus
197. Shamonda virus
198. Sango virus
199. Tamana bat virus
200. Tioman virus
201. Human metapneumovirus
202. Wesselsbron virus
203. Rotavirus F chicken/03V0568/DEU/2003
204. Rotavirus G
205. Rotavirus G chicken/03V0567/DEU/2003
206. Zaire ebolavirus
207. Reston ebolavirus
208. Sudan ebolavirus
209. Cote d'Ivoire ebolavirus
210. Bear Canyon virus
211. Simian T-lymphotropic virus 1
212. Human T-lymphotropic virus 2
213. Simian T-lymphotropic virus 3
214. Human parechovirus 1
215. Great Island virus
216. Tribec virus

217. Kemerovo virus
218. Cardiovirus D/VI2223/2004
219. Goose paramyxovirus SF02
220. Toscana virus
221. Cupixi virus
222. Aravan virus
223. Kamiti River virus
224. Mossman virus
225. Liao ning virus
226. Irkut virus
227. Thottapalayam virus
228. Human coronavirus NL63
229. Human parvovirus 4
230. Human coronavirus HKU1
231. Mopeia Lassa virus reassortant 29
232. Human T-lymphotropic virus 4
233. J-virus
234. Human bocavirus
235. Human papillomavirus type 11
236. Human papillomavirus type 43
237. Human papillomavirus - 54
238. Human papillomavirus type 114
239. Human papillomavirus type 120
240. Human papillomavirus type 4
241. Human papillomavirus type 48
242. Human papillomavirus type 50
243. Human papillomavirus type 60
244. Human papillomavirus type 88
245. Human papillomavirus - 1
246. Human papillomavirus type 63
247. Human papillomavirus type 41
248. Human papillomavirus type 75
249. Human papillomavirus type 92
250. Human papillomavirus SIBX1
251. Human papillomavirus type 28
252. Human papillomavirus type 16
253. Human papillomavirus type 39
254. Human papillomavirus type 27
255. Human papillomavirus type 82
256. Human papillomavirus type 42
257. Human papillomavirus type 34
258. Human papillomavirus type 66
259. Human papillomavirus type 19
260. Beilong virus
261. Culex flavivirus
262. Aedes flavivirus
263. Small anellovirus 1
264. KI polyomavirus
265. WU Polyomavirus
266. Human rhinovirus C
267. Merkel cell polyomavirus
268. Chapare virus
269. Human adenovirus 52
270. Bundibugyo ebolavirus
271. Morogoro virus
272. Human bocavirus 2
273. Human cosavirus B
274. Human cosavirus D
275. Human cosavirus E
276. Nariva virus
277. Human enteric coronavirus strain 4408
278. Candiru virus
279. Echarate virus
280. Chaoyang virus
281. Quang Binh virus
282. Lujo virus
283. Mosquito flavivirus
284. Avian bornavirus
285. Torque teno virus 1
286. Torque teno virus 2
287. Torque teno virus 3
288. Torque teno virus 4
289. Torque teno virus 6
290. Torque teno virus 7
291. Torque teno virus 8
292. Torque teno virus 10
293. Torque teno virus 12
294. Torque teno virus 14
295. Torque teno virus 15
296. Torque teno virus 16
297. Torque teno virus 19
298. Torque teno virus 25
299. Torque teno virus 26
300. Torque teno virus 27
301. Torque teno virus 28
302. Torque teno mini virus 1
303. Torque teno mini virus 2
304. Torque teno mini virus 3
305. Torque teno mini virus 4
306. Torque teno mini virus 5
307. Torque teno mini virus 6
308. Torque teno mini virus 7
309. Torque teno mini virus 8
310. Torque teno mini virus 9
311. Torque teno midi virus 1
312. Torque teno midi virus 2
313. Torque teno tamarin virus
314. Torque teno douroucouli virus
315. Torque teno felis virus
316. Torque teno canis virus
317. Torque teno sus virus 1a
318. Human coronavirus OC43
319. SARS coronavirus
320. Luna virus
321. Arumowot virus
322. Human papillomavirus type 134
323. Human papillomavirus type 129
324. Human papillomavirus type 90
325. Human polyomavirus 9
326. Donggang virus
327. Human gyrovirus type 1
328. Seal anellovirus TFFN/USA/2006

329. Severe fever with thrombocytopenia syndrome virus
330. SFTS virus JS4
331. FTLS virus
332. Ixcanal virus
333. Durania virus
334. Cutthroat trout virus
335. Human papillomavirus type 136
336. Human papillomavirus type 140
337. Lunk virus NKS-1
338. Ikoma lyssavirus
339. Macaca fascicularis papillomavirus 2
340. Human papillomavirus 101
341. Human papillomavirus 109
342. Human papillomavirus type 119
343. Human papillomavirus 116
344. Human papillomavirus type 130
345. Bat sapovirus TLC58/HK
346. Human papillomavirus type 154
347. MW polyomavirus
348. Bat hepevirus
349. Heartland virus
350. Torque teno sus virus k2
351. Torque teno zalophus virus 1
352. CAS virus
353. Golden Gate virus
354. Human cosavirus
355. Human astrovirus
356. Rodent hepacivirus
357. Human polyomavirus 12
358. Human papillomavirus type 132
359. Human cosavirus A2
360. Middle East respiratory syndrome coronavirus
361. Human cyclovirus VS5700009
362. Adult diarrheal rotavirus strain J19
363. Brazoran virus
364. Razdan virus
365. Equine hepacivirus JPN3/JAPAN/2013
366. Human papillomavirus type 167
367. Seal anellovirus 3
368. Seal anellovirus 2
369. Human cyclovirus
370. Human papillomavirus
371. Human papillomavirus type 178
372. Human bocavirus 3
373. Human parvovirus B19
374. Lloviu virus
375. Human papillomavirus type 126
376. Human papillomavirus type 199
377. Human papillomavirus type 128
378. Human papillomavirus type 131
379. Human papillomavirus type 135
380. Human papillomavirus type 137
381. Human papillomavirus type 144
382. Human papillomavirus type 166
383. Human astrovirus BF34
384. Human circovirus VS6600022
385. Reptile bornavirus 1
386. Torque teno Tadarida brasiliensis virus

Supplemental Information S2: A list of human viruses that can not be covered by the list of 88 8-mer oligonucleotides, a total of 59.

1. MW_polyomavirus
2. Human_immunodeficiency_virus_1
3. Human_bocavirus_4_NI_strain_HBoV4-NI-385
4. Influenza_C_virus
5. Human_papillomavirus_type_50
6. Simian_virus_40
7. Torque_teno_mini_virus_9
8. BK_polyomavirus
9. Human_erythrovirus_V9
10. Pirital_virus
11. Guanarito_virus_segment
12. Hepatitis_delta_virus
13. Mopeia_Lassa_reassortant_29
14. _Mopeia_virus_AN20410
15. Simian_T-lymphotropic_virus_2
16. Torque_teno_virus_1
17. Small_anellovirus_1
18. Small_anellovirus_2
19. Tick-borne_encephalitis_virus
20. Louping_ill_virus
21. Langat_virus
22. Modoc_virus
23. Tamana_bat_virus
24. Human_parvovirus_4
25. Human_papillomavirus_type_90
26. Hepatitis_B_virus
27. JC_polyomavirus
28. Human_papillomavirus_type_26
29. Human_papillomavirus_type_34,
30. Avian_metapneumovirus
31. Mobala_virus
32. WU_Polyomavirus
33. Oliveros_virus
34. Merkel_cell_polyomavirus
35. Thottapalayam_virus
36. California_sea_lion_anellovirus
37. Torque_teno_mini_virus_8
38. Torque_teno_virus_4
39. Torque_teno_sus_virus_1
40. Torque_teno_canis_virus
41. Torque_teno_felis_virus
42. Torque_teno_virus_28
43. Torque_teno_virus_12
44. Torque_teno_virus_10
45. Torque_teno_mini_virus_2
46. Torque_teno_douroucouli_virus
47. Torque_teno_mini_virus_3
48. Torque_teno_mini_virus_4
49. Torque_teno_mini_virus_6
50. Torque_teno_virus_15
51. Torque_teno_virus_2
52. Human_polyomavirus_9
53. Seal_anellovirus_TFFN/USA/2006
54. Candiru_virus_segment
55. Torque_teno_virus
56. Luna_virus
57. TTV-like_mini_virus_isolate_TTMV_LY1
58. Human_TMEV-like_cardiovirus
59. Human_polyomavirus_12_strain_hu1403

Supplemental Information S3: A clinical variant of *Streptococcus pneumonia* (above) identified by PATHseq method. *Streptococcus mitis* (below) is shown as a control. Both *Streptococci* grow on blood agar plate.

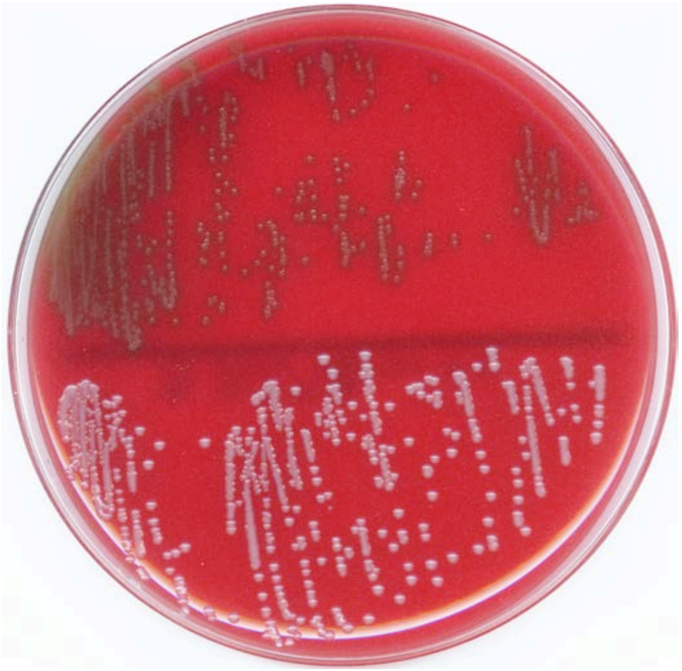

Supplemental Information S4: Antimicrobial susceptibility testing showed that this isolated variant of *Streptococcus pneumonia* was sensitive to cefotaxime, chloramphenicol, oxacillin, penicillin, tetracycline, and vancomycin, but resistant to erythromycin and ethyl hydrocupreine, and partially resistant to sulfamethoxazole trimethoprim.

Abbr.	Antibiotics Discs	Unit	*S. pneumo*
CTX30	Cefotaxime	30ug	+
C30	Chloramphenicol	30ug	+
OX1	Oxacillin	1ug	+
P10	Penicillin	10IU	+
TE30	Tetracyclin	30ug	+
Va30	Vancomycin	30ug	+
E15	Erythromycin	15ug	-
P	Etyl hydrocupreine HCl	5.0ug	-
SXT	Sulfamethoxazole/Trimethoprim	23.75/1.25ug	+/-

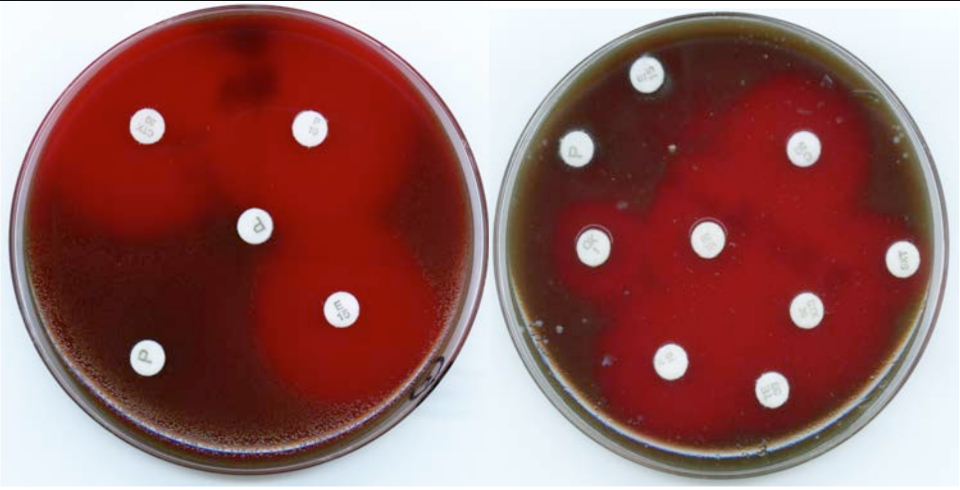

Supplemental Information S5: The metabolic biochemical details detected by VITEC 2 Systems Version 05.04 of *S. pneumonia* identified by PATHseq method

Well	Abbr.	*S. peumo*	Well	Abbr.	*S. peumo*	Well	Abbr.	*S. peumo*	Well	Abbr.	*S. peumo*
2	AMY	-	4	PIPLC	-	5	dXYL	-	8	ADH1	-
9	BGAL	+	11	AGLU	+	13	APPA	+	14	CDEX	-
15	AspA	-	16	BGAR	+	17	AMAN	-	19	PHOS	-
20	LeuA	+	23	ProA	-	24	BGURr	-	25	AGAL	+
26	PyrA	+	27	BGUR	-	28	AlaA	+	29	TyrA	+
30	dSOR	-	31	URE	-	32	POLYB	+	37	dGAL	+
38	dRIB	-	39	ILATk	-	42	LAC	+	44	NAG	+
45	dMAL	+	46	BACI	-	47	NOVO	-	50	NC6.5	-
52	dMAN	-	53	dMNE	+	54	MBdG	-	56	PUL	-
57	dRAF	+	58	O129R	-	59	SAL	-	60	SAC	+
62	dTRE	+	63	ADH2s	-	64	OPTO	-			

Abbreviation: ***AMY:*** *D-AMYGDALIN;* ***PIPLC:*** *PHOSPHATIDYLINOSITOL PHOSPHOLIPASE C****;*** **dXYL:** D-XYLOSE; ***ADH1:*** *ARGININE DIHYDROLASE 1;* ***BGAL:*** *BETA-GALACTOSIDASE;* ***AGLU:*** *ALPHA-GLUCOSIDASE;* ***APPA:*** *Ala-Phe-Pro-ARYLAMIDASE;* ***CDEX:*** *CYCLODEXTRIN;* ***AspA:*** *L-Aspartate ARYLAMIDASE;* ***BGAR:*** *BETA GALACTOPYRANOSIDASE****; AMAN:*** *ALPHA-MANNOSIDASE;* ***PHOS:*** *PHOSPHATASE****; LeuA:*** *Leucine ARYLAMIDASE;* ***ProA:*** *L-Proline ARYLAMIDASE;* ***BGURr:*** *BETA-GLUCURONIDASE;* ***AGAL:*** *ALPHAGALACTOSIDASE;* ***PyrA:*** *L-Pyrrolidonyl-ARYLAMIDASE;* ***BGUR:*** *BETA-GLUCURONIDASE;* ***AlaA:*** *Alanine ARYLAMIDASE;* ***TyrA:*** *Tyrosine ARYLAMIDASE****; dSOR:*** *D-SORBITOL;* ***URE:*** *UREASE;* ***POLYB:*** *POLYMIXIN B RESISTANCE****; dGAL:*** *D-GALACTOSE;* ***dRIB:*** *D-RIBOSE;* ***ILATk:*** *L-LACTATE alkalinization****; LAC:*** *LACTOSE;* ***NAG:*** *NACETYL-D-GLUCOSAMINE****; dMAL:*** *D-MALTOSE;* ***BACI:*** *BACITRACIN RESISTANCE;* ***NOVO:*** *NOVOBIOCIN RESISTANCE;* ***NC6.5:*** *GROWTH IN 6.5% NaCl;* ***dMAN:*** *D-MANNITOL;* ***dMNE:*** *D-MANNOSE;* ***MBdG:*** *METHYL-B-DGLUCOPYRANOSIDE;* ***PUL:*** *PULLULAN;* ***dRAF:*** *D-RAFFINOSE;* ***O129R:*** *O/129 RESISTANCE (comp.vibrio);* ***SAL:*** *SALICIN;* ***SAC:*** *SACCHAROSE/SUCROSE;* ***dTRE:*** *D-TREHALOSE;* ***ADH2s:*** *ARGININE DIHYDROLASE 2;* ***OPTO:*** *OPTOCHIN RESISTANCE*

Supplemental Information S6: PCR assay of housekeeping genes for *S. pneumonia.* Lane 1: pneumolysin (ply) gene; Lane 2: major autolysin (lytA); Lane 3: atypical autolysin (lytA101) gene; Lane M: 1 kb DNA ladder.

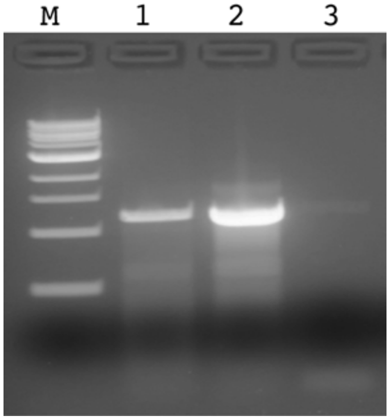

Supplemental Information S7: Sequences of 7-mer oligonucleotides that do not match the sequences of top 1,000 human transcripts

Non-match to all of the bases.
[6540] ATAGCGC
Number of missing fragments: 1

Non-match to the 3' 1000 bases.
[4719] TTGCGAC
[6540] ATAGCGC
Number of missing fragments: 2

Non-match to the 3' 500 bases.
[582] GCACGAA
[632] AGTCGAA
[921] CGCGTAA
[1182] GTCGACA
[1603] TAACGCA
[2425] CGTCCGA
[2457] CGCGCGA
[2493] CTTGCGA
[2531] TAGTCGA
[3302] GCGTATA
[3561] CGGTCTA
[3577] CGTTCTA
[3651] TAACGTA
[3683] TAGCGTA
[3684] ACGCGTA
[3692] ATGCGTA
[3705] CGTCGTA
[3877] CCGATTA
[4684] ATACGAC
[4719] TTGCGAC
[4902] GCGATAC
[6372] ACGTAGC
[6374] GCGTAGC
[6377] CGGTAGC
[6414] GTAACGC
[6462] GTTACGC
[6540] ATAGCGC
[6559] TTCGCGC
[7321] CGCGATC
[7398] GCGTATC
[8807] TCGCGAG
[9017] CGTATAG
[9113] CGCGTAG
[9126] GCGGTAG
[9225] CGAAACG
[9271] TCTAACG
[9356] ATAGACG
[9464] AGTTACG
[9465] CGTTACG
[9507] TAGACCG
[9667] TAATCCG
[9783] TCTAGCG
[9801] CGACGCG
[9827] TAGCGCG
[9836] ATGCGCG
[9840] AATCGCG
[9854] GTTCGCG
[9855] TTTCGCG
[9980] ATTTGCG
[9987] TAAATCG
[10124] ATAGTCG
[10211] TAGTTCG
[10691] TAATCGG
[12703] TTCGCAT
[12900] ACGCGAT
[12921] CGTCGAT
[13081] CGCATAT
[13200] AACGTAT
[13209] CGCGTAT
[14606] GTAACGT
[14735] TTAGCGT
[14748] ATCGCGT
[14793] CGATCGT
[15929] CGTAGTT
[15993] CGTCGTT
Number of missing fragments: 65

Supplemental Information S8: Sequences of 8-mer oligonucleotides that do not match the sequences of top 2,000 human transcripts

Non-match to full sequences:
[217] CGCTAAAA
[3108] ACGAATAA
[3651] TAACGTAA
[6540] ATAGCGCA
[9789] CTTAGCGA
[9792] AAACGCGA
[10127] TTAGTCGA
[10153] CGGGTCGA
[12688] AACGCATA
[12879] TTACGATA
[12900] ACGCGATA
[12908] ATGCGATA
[13209] CGCGTATA
[13939] TATCGCTA
[14537] CGATAGTA
[14565] CCGTAGTA
[14569] CGGTAGTA
[14601] CGAACGTA
[14607] TTAACGTA
[14630] GCGACGTA
[14642] GATACGTA
[14670] GTACCGTA
[14745] CGCGCGTA
[14793] CGATCGTA
[14960] AATCGGTA
[14963] TATCGGTA
[15940] ACACGTTA
[15980] ATGCGTTA
[16020] ACCGGTTA
[16025] CGCGGTTA
[17161] CGAATAAC
[17319] TCGGTAAC
[18803] TATCCGAC
[18876] ATTGCGAC
[19059] TATCGGAC
[19609] CGCGATAC
[20041] CGACGTAC
[20044] ATACGTAC
[20067] TAGCGTAC
[23753] CGATATCC
[24809] CGGTAAGC
[25648] AATAACGC
[25715] TATCACGC
[26163] TATAGCGC
[29497] CGTATATC
[29593] CGCGTATC
[31347] TATCGGTC
[35299] TAGTCGAG
[35879] TCGAATAG
[37070] GTATAACG
[37077] CCCTAACG
[37091] TAGTAACG
[37645] CTAATACG
[37820] ATTGTACG
[37924] ACGAACCG
[38691] TAGATCCG
[38971] TGTAAGCG
[39119] TTATAGCG
[39230] GTTACGCG
[39299] TAAGCGCG
[39308] ATAGCGCG
[39361] CAATCGCG
[39375] TTATCGCG
[40143] TTATATCG
[40483] TAGAGTCG
[40579] TAAGGTCG
[40752] AATATTCG
[40761] CGTATTCG
[41764] ACGATAGG
[49763] TAGCGAAT
[49785] CGTCGAAT
[50073] CGCGTAAT
[50085] CCGGTAAT
[51433] CGGTAGAT
[51687] TCGTCGAT
[51689] CGGTCGAT
[52249] CGCAATAT
[52262] GCGAATAT
[52835] TAGCGTAT
[52839] TCGCGTAT
[58563] TAATACGT
[58940] ATTAGCGT
[59184] AATATCGT
[59593] CGATAGGT
[59724] ATACCGGT
[59875] TAGTCGGT
[61670] GCGTAATT
[63811] TAACCGTT
Number of missing fragments: 88

Non-match to the 3' 1000 bases:
[217] CGCTAAAA
[857] CGCCTAAA
[870] GCGCTAAA
[871] TCGCTAAA
[1254] GCGTACAA
[1612] ATACGCAA
[1651] TATCGCAA
[1656] AGTCGCAA
[2462] GTCGCGAA
[2468] ACGGCGAA
[2505] CGATCGAA
[3108] ACGAATAA
[3177] CGGCATAA
[3300] ACGTATAA
[3302] GCGTATAA
[3305] CGGTATAA
[3367] TCGACTAA
[3401] CGACCTAA
[3481] CGCGCTAA
[3484] ATCGCTAA
[3492] ACGGCTAA
[3557] CCGTCTAA
[3561] CGGTCTAA
[3651] TAACGTAA
[3658] GGACGTAA
[3667] TACCGTAA
[3687] TCGCGTAA
[3692] ATGCGTAA
[3710] GTTCGTAA
[3739] TGCGGTAA
[3743] TTCGGTAA
[3877] CCGATTAA
[3913] CGACTTAA
[3998] GTCGTTAA
[4329] CGGTAACA
[4729] CGTCGACA
[4903] TCGATACA
[5699] TAACGCCA
[6540] ATAGCGCA
[6579] TATGCGCA
[7113] CGATTGCA
[7460] ACGACTCA
[7747] TAACGTCA
[8425] CGGTAAGA
[9113] CGCGTAGA
[9127] TCGGTAGA
[9271] TCTAACGA
[9336] AGTCACGA
[9357] CTAGACGA
[9367] TCCGACGA
[9411] TAATACGA
[9700] ACGTCCGA
[9785] CGTAGCGA
[9789] CTTAGCGA
[9792] AAACGCGA
[9830] GCGCGCGA
[9843] TATCGCGA
[9853] CTTCGCGA
[9854] GTTCGCGA
[9977] CGTTGCGA
[9997] CTAATCGA
[10025] CGGATCGA
[10041] CGTATCGA
[10083] TAGCTCGA
[10084] ACGCTCGA
[10124] ATAGTCGA
[10127] TTAGTCGA
[10130] GACGTCGA
[10142] GTCGTCGA
[10150] GCGGTCGA
[10153] CGGGTCGA
[10173] CTTGTCGA
[10211] TAGTTCGA
[10224] AATTTCGA
[10691] TAATCGGA
[10723] TAGTCGGA
[12345] CGTAAATA
[12434] GACGAATA
[12647] TCGCCATA
[12688] AACGCATA
[12692] ACCGCATA
[12696] AGCGCATA
[12838] GCGAGATA
[12873] CGACGATA
[12879] TTACGATA
[12900] ACGCGATA
[12905] CGGCGATA
[12908] ATGCGATA
[12921] CGTCGATA
[13033] CGGTGATA
[13113] CGTATATA
[13161] CGGCTATA
[13209] CGCGTATA
[13222] GCGGTATA
[13223] TCGGTATA
[13225] CGGGTATA
[13769] CGATCCTA
[13939] TATCGCTA
[14537] CGATAGTA
[14565] CCGTAGTA
[14566] GCGTAGTA
[14569] CGGTAGTA
[14601] CGAACGTA
[14607] TTAACGTA
[14630] GCGACGTA
[14642] GATACGTA
[14670] GTACCGTA
[14671] TTACCGTA
[14733] CTAGCGTA
[14734] GTAGCGTA
[14745] CGCGCGTA
[14748] ATCGCGTA
[14752] AAGGCGTA

[14756] ACGGCGTA
[14793] CGATCGTA
[14947] TAGCGGTA
[14956] ATGCGGTA
[14960] AATCGGTA
[14963] TATCGGTA
[14971] TGTCGGTA
[14985] CGAGGGTA
[15509] CCCGATTA
[15625] CGAACTTA
[15940] ACACGTTA
[15979] TGGCGTTA
[15980] ATGCGTTA
[15982] GTGCGTTA
[16020] ACCGGTTA
[16025] CGCGGTTA
[16073] CGATGTTA
[16537] CGCGAAAC
[16633] CGTTAAAC
[16649] CGAACAAC
[16999] TCGCGAAC
[17007] TTGCGAAC
[17161] CGAATAAC
[17188] ACGATAAC
[17298] GACGTAAC
[17299] TACGTAAC
[17305] CGCGTAAC
[17319] TCGGTAAC
[17381] CCGTTAAC
[17676] ATAACCAC
[18019] TAGCGCAC
[18044] ATTCGCAC
[18665] CGGTAGAC
[18739] TATACGAC
[18803] TATCCGAC
[18819] TAAGCGAC
[18847] TTCGCGAC
[18866] GATGCGAC
[18876] ATTGCGAC
[18879] TTTGCGAC
[18883] TAATCGAC
[18928] AATTCGAC
[19059] TATCGGAC
[19609] CGCGATAC
[19689] CGGTATAC
[20025] CGTAGTAC
[20041] CGACGTAC
[20044] ATACGTAC
[20062] GTCCGTAC
[20067] TAGCGTAC
[20089] CGTCGTAC
[20711] TCGTAACC
[20729] CGTTAACC
[21084] ATCCGACC
[21116] ATTCGACC
[22931] TACGCGCC
[23753] CGATATCC
[24809] CGGTAAGC
[25382] GCGATAGC
[25609] CGAAACGC
[25648] AATAACGC
[25661] CTTAACGC
[25715] TATCACGC
[25759] TTCGACGC
[25803] TGATACGC
[25857] CAAACCGC
[25891] TAGACCGC
[25923] TAACCCGC
[26051] TAATCCGC
[26147] TAGAGCGC
[26162] GATAGCGC
[26163] TATAGCGC
[26175] TTTAGCGC
[26188] ATACGCGC
[26319] TTATGCGC
[26355] TATTGCGC
[26422] GCTATCGC
[26508] ATAGTCGC
[26572] ATATTCGC
[27251] TATCGGGC
[28307] TACGGTGC
[28818] GACGAATC
[28890] GGCTAATC
[29305] CGTCGATC
[29497] CGTATATC
[29593] CGCGTATC
[30988] ATAACGTC
[31043] TAACCGTC
[31181] CTATCGTC
[31203] TAGTCGTC
[31289] CGTAGGTC
[31310] GTACGGTC
[31347] TATCGGTC
[32329] CGACGTTC
[33593] CGTATAAG
[33673] CGAGTAAG
[33700] ACGGTAAG
[35231] TTCGCGAG
[35299] TAGTCGAG
[35879] TCGAATAG
[35943] TCGCATAG
[35985] CACGATAG
[35986] GACGATAG
[35991] TCCGATAG
[35993] CGCGATAG
[35996] ATCGATAG
[36148] ACTACTAG
[36151] TCTACTAG
[36416] AAACGTAG
[36452] ACGCGTAG
[36454] GCGCGTAG
[36508] ATCGGTAG
[36509] CTCGGTAG
[36633] CGCATTAG
[36665] CGTATTAG
[36745] CGAGTTAG
[36761] CGCGTTAG
[36774] GCGGTTAG
[36837] CCGTTTAG
[36901] CCGAAACG
[36922] GGTAAACG
[37017] CGCGAACG
[37019] TGCGAACG
[37028] ACGGAACG
[37065] CGATAACG
[37070] GTATAACG
[37071] TTATAACG
[37077] CCCTAACG
[37086] GTCTAACG
[37088] AAGTAACG
[37091] TAGTAACG
[37102] GTGTAACG
[37112] AGTTAACG
[37182] GTTACACG
[37264] AACGCACG
[37428] ACTAGACG
[37456] AACCGACG
[37487] TTGCGACG
[37496] AGTCGACG
[37503] TTTCGACG
[37636] ACAATACG
[37645] CTAATACG
[37647] TTAATACG
[37676] ATGATACG
[37688] AGTATACG
[37783] TCCGTACG
[37784] AGCGTACG
[37788] ATCGTACG
[37799] TCGGTACG
[37820] ATTGTACG
[37849] CGCTTACG
[37875] TATTTACG
[37924] ACGAACCG
[37938] GATAACCG
[37951] TTTAACCG
[38086] GCATACCG
[38156] ATAACCCG
[38183] TCGACCCG
[38451] TATAGCCG
[38464] AAACGCCG
[38691] TAGATCCG
[38799] TTAGTCCG
[38971] TGTAAGCG
[39112] AGATAGCG
[39113] CGATAGCG
[39119] TTATAGCG
[39134] GTCTAGCG
[39183] TTAACGCG
[39207] TCGACGCG
[39219] TATACGCG
[39230] GTTACGCG
[39251] TACCCGCG
[39299] TAAGCGCG
[39308] ATAGCGCG
[39356] ATTGCGCG
[39361] CAATCGCG
[39363] TAATCGCG
[39373] CTATCGCG
[39375] TTATCGCG
[39410] GATTCGCG
[39411] TATTCGCG
[39436] ATAAGGCG
[39539] TATCGGCG
[39738] GGTATGCG
[39820] ATAGTGCG
[39881] CGATTGCG
[39887] TTATTGCG
[39911] TCGTTGCG
[39922] GATTTGCG
[39975] TCGAATCG
[39998] GTTAATCG
[40081] CACGATCG
[40130] GAATATCG
[40135] TCATATCG
[40143] TTATATCG
[40160] AAGTATCG
[40183] TCTTATCG
[40334] GTAGCTCG
[40335] TTAGCTCG
[40348] ATCGCTCG
[40385] CAATCTCG
[40457] CGAAGTCG
[40483] TAGAGTCG
[40496] AATAGTCG
[40503] TCTAGTCG
[40515] TAACGTCG
[40527] TTACGTCG
[40548] ACGCGTCG
[40579] TAAGGTCG
[40595] TACGGTCG
[40596] ACCGGTCG
[40692] ACTTGTCG
[40752] AATATTCG
[40761] CGTATTCG
[40764] ATTATTCG
[40845] CTAGTTCG
[40847] TTAGTTCG
[40862] GTCGTTCG
[40935] TCGTTTCG
[41555] TACCGAGG
[41764] ACGATAGG
[41765] CCGATAGG
[41961] CGGTTAGG
[42084] ACGCACGG

[42254] GTAACCGG
[42691] TAATGCGG
[42800] AATATCGG
[42892] ATAGTCGG
[42894] GTAGTCGG
[42895] TTAGTCGG
[42899] TACGTCGG
[42910] GTCGTCGG
[42916] ACGGTCGG
[42959] TTATTCGG
[42979] TAGTTCGG
[43404] ATAGCGGG
[45833] CGAATATG
[47561] CGATCGTG
[48935] TCGATTTG
[49743] TTACGAAT
[49763] TAGCGAAT
[49785] CGTCGAAT
[49808] AACGGAAT
[49957] CCGATAAT
[50073] CGCGTAAT
[50078] GTCGTAAT
[50085] CCGGTAAT
[50121] CGATTAAT
[50150] GCGTTAAT
[50324] ACCGACAT
[50409] CGGTACAT
[50755] TAACGCAT
[50761] CGACGCAT
[51433] CGGTAGAT
[51596] ATAGCGAT
[51605] CCCGCGAT
[51621] CCGGCGAT
[51649] CAATCGAT
[51669] CCCTCGAT
[51671] TCCTCGAT
[51685] CCGTCGAT
[51687] TCGTCGAT
[51689] CGGTCGAT
[51697] CATTCGAT
[51833] CGTCGGAT
[52249] CGCAATAT
[52262] GCGAATAT
[52324] ACGCATAT
[52377] CGCGATAT
[52457] CGGTATAT
[52489] CGAACTAT
[52835] TAGCGTAT
[52839] TCGCGTAT
[52840] AGGCGTAT
[52850] GATCGTAT
[52859] TGTCGTAT
[52901] CCGGGTAT
[53095] TCGCTTAT
[53148] ATCGTTAT
[53401] CGCGAACT
[53860] ACGCGACT
[54170] GGCGTACT
[55497] CGATAGCT
[55545] CGTTAGCT
[55614] GTTACGCT
[56723] TACGCTCT
[57497] CGCGAAGT
[57657] CGTACAGT
[58258] GACGTAGT
[58260] ACCGTAGT
[58265] CGCGTAGT
[58267] TGCGTAGT
[58340] ACGTTAGT
[58526] GTCGACGT
[58535] TCGGACGT
[58563] TAATACGT
[58617] CGTTACGT
[58620] ATTTACGT
[58628] ACAACCGT
[58636] ATAACCGT
[58659] TAGACCGT
[58739] TATCCCGT
[58937] CGTAGCGT
[58940] ATTAGCGT
[58951] TCACGCGT
[58953] CGACGCGT
[58988] ATGCGCGT
[59043] TAGGGCGT
[59047] TCGGGCGT
[59173] CCGATCGT
[59184] AATATCGT
[59203] TAACTCGT
[59278] GTAGTCGT
[59280] AACGTCGT
[59305] CGGGTCGT
[59593] CGATAGGT
[59662] GTAACGGT
[59704] AGTACGGT
[59705] CGTACGGT
[59715] TAACCGGT
[59724] ATACCGGT
[59788] ATAGCGGT
[59841] CAATCGGT
[59843] TAATCGGT
[59875] TAGTCGGT
[61641] CGATAATT
[61670] GCGTAATT
[61849] CGCGCATT
[62032] AACCGATT
[62036] ACCCGATT
[62052] ACGCGATT
[62329] CGTCTATT
[62365] CTCGTATT
[63811] TAACCGTT
[63856] AATCCGTT
[63884] ATAGCGTT
[63890] GACGCGTT
[63975] TCGTCGTT
[63977] CGGTCGTT
[63993] CGTTCGTT
[64091] TGCCGGTT
[65433] CGCGTTTT
Number of missing fragments: 455

Non-match to the 3' 500 bases:
[3651] TAACGTA
[3684] ACGCGTA
[9113] CGCGTAG
[14793] CGATCGT
Number of missing fragments: 4

Supplemental Information S9: Sequences of 8-mer oligonucleotides that do not match the sequences of top 4,000 human transcripts

Non-match to all of the bases:
[14745] CGCGCGTA
Number of missing fragments: 1

Non-match to the 3' 1000 bases:
[3302] GCGTATAA
[3739] TGCGGTAA
[9700] ACGTCCGA
[9785] CGTAGCGA
[9853] CTTCGCGA
[9977] CGTTGCGA
[10124] ATAGTCGA
[10127] TTAGTCGA
[12434] GACGAATA
[12688] AACGCATA
[13209] CGCGTATA
[13222] GCGGTATA
[13939] TATCGCTA
[14537] CGATAGTA
[14601] CGAACGTA
[14642] GATACGTA
[14745] CGCGCGTA
[14793] CGATCGTA
[15980] ATGCGTTA
[16537] CGCGAAAC
[17305] CGCGTAAC
[18019] TAGCGCAC
[18665] CGGTAGAC
[18739] TATACGAC
[18803] TATCCGAC
[18847] TTCGCGAC
[19689] CGGTATAC
[20729] CGTTAACC
[21084] ATCCGACC
[25648] AATAACGC
[26163] TATAGCGC
[26175] TTTAGCGC
[26188] ATACGCGC
[26572] ATATTCGC
[31203] TAGTCGTC
[35299] TAGTCGAG
[35879] TCGAATAG
[35943] TCGCATAG
[37017] CGCGAACG
[37019] TGCGAACG
[37676] ATGATACG
[37688] AGTATACG
[37784] AGCGTACG
[37788] ATCGTACG
[37924] ACGAACCG
[38799] TTAGTCCG
[39183] TTAACGCG
[39299] TAAGCGCG
[39373] CTATCGCG
[39411] TATTCGCG
[39539] TATCGGCG
[39881] CGATTGCG
[40496] AATAGTCG
[40527] TTACGTCG
[40595] TACGGTCG
[40596] ACCGGTCG
[40761] CGTATTCG
[40845] CTAGTTCG
[40847] TTAGTTCG
[49763] TAGCGAAT
[49785] CGTCGAAT
[50073] CGCGTAAT
[51687] TCGTCGAT
[51689] CGGTCGAT
[52839] TCGCGTAT
[54170] GGCGTACT
[58260] ACCGTAGT
[58937] CGTAGCGT
[59173] CCGATCGT
[59593] CGATAGGT
[59704] AGTACGGT
[59724] ATACCGGT
[62052] ACGCGATT
[63993] CGTTCGTT
Number of missing fragments: 74

Non-match to the 3' 500 bases:
[14793] CGATCGT
Number of missing fragments: 1

Supplemental Information S10: Sequences of 9-mer oligonucleotides that do not match the sequences of all 86,248 human transcripts

Non-match to all of the bases:
[51615] TTCGCGATA
Number of missing fragments: 1

Non-match to the 3' 1000 bases:
[13721] CGCGCCTAA
[14793] CGATCGTAA
[14948] ACGCGGTAA
[49764] ACGCGAATA
[50791] TCGCGCATA
[51615] TTCGCGATA
[58265] CGCGTAGTA
[58959] TTACGCGTA
[66457] CGCGTAAAC
[148425] CGATTAACG
[151143] TCGCGTACG
[159527] TCGATTGCG
[162061] CTAACGTCG
[200292] ACGCGTAAT
[200295] TCGCGTAAT
[206759] TCGGTCGAT
[207161] CGTACGGAT
[236003] TAGTCGCGT
[246169] CGCGCAATT
[255206] GCGTACGTT
Number of missing fragments: 20

Non-match to the 3' 500 bases:
[2462] GTCGCGAAA
[6564] ACGGCGCAA
[7780] ACGCGTCAA
[9831] TCGCGCGAA
[10022] GCGATCGAA
[10215] TCGTTCGAA
[13721] CGCGCCTAA
[13927] TCGCGCTAA
[14743] TCCGCGTAA
[14744] AGCGCGTAA
[14793] CGATCGTAA
[14948] ACGCGGTAA
[25758] GTCGACGCA
[25831] TCGTACGCA
[37020] ATCGAACGA
[37264] AACGCACGA
[37495] TCTCGACGA
[37657] CGCATACGA
[39207] TCGACGCGA
[39218] GATACGCGA
[39299] TAAGCGCGA
[39309] CTAGCGCGA
[39324] ATCGCGCGA
[40339] TACGCTCGA
[40515] TAACGTCGA
[40526] GTACGTCGA
[49764] ACGCGAATA
[49765] CCGCGAATA
[50278] GCGCACATA
[50329] CGCGACATA
[50791] TCGCGCATA
[51465] CGAACGATA
[51481] CGCACGATA
[51557] CCGCCGATA
[51615] TTCGCGATA
[51689] CGGTCGATA
[52838] GCGCGTATA
[53145] CGCGTTATA
[54162] GACGTACTA
[56980] ACCGGTCTA
[58265] CGCGTAGTA
[58409] CGGAACGTA
[58534] GCGGACGTA
[58665] CGGACCGTA
[58937] CGTAGCGTA
[58959] TTACGCGTA
[58974] GTCCGCGTA
[58990] GTGCGCGTA
[58995] TATCGCGTA
[59001] CGTCGCGTA
[59005] CTTCGCGTA
[59292] ATCGTCGTA
[59301] CCGGTCGTA
[59687] TCGACGGTA
[59721] CGACCGGTA
[62053] CCGCGATTA
[64665] CGCGATTTA
[65126] GCGCGTTTA
[66150] GCGCGAAAC
[66457] CGCGTAAAC
[67890] GATACGAAC
[68041] CGATCGAAC
[75331] TAACGCGAC
[75671] TCCGTCGAC
[80231] TCGCCGTAC
[91193] CGTAACGCC
[91715] TAACGCGCC
[101529] CGCGATAGC
[102629] CCGTAACGC
[102630] GCGTAACGC
[102979] TAACGACGC
[102988] ATACGACGC
[103374] GTATTACGC
[104717] CTAACGCGC
[104761] CGTACGCGC
[104766] GTTACGCGC
[104931] TAGTCGCGC
[104947] TATTCGCGC
[105020] ATTAGGCGC
[106099] TATCGTCGC
[106126] GTAGGTCGC
[106468] ACGTTTCGC
[115609] CGCGTAATC
[118217] CGATCTATC
[124493] CTACGCGTC
[129241] CGCTAGTTC
[141203] TACGTCGAG
[143974] GCGCGATAG
[144537] CGCGACTAG
[144614] GCGTACTAG
[148083] TATCGAACG
[148265] CGGATAACG
[148327] TCGCTAACG
[148377] CGCGTAACG
[148425] CGATTAACG
[148457] CGGTTAACG
[149753] CGTTAGACG
[149901] CTAGCGACG
[149968] AACTCGACG
[150630] GCGCATACG
[150985] CGATCTACG
[151116] ATACGTACG
[151123] TACCGTACG
[151138] GAGCGTACG
[151143] TCGCGTACG
[151181] CTAGGTACG
[151709] CTCGAACCG
[151710] GTCGAACCG
[152345] CGCATACCG
[153200] AATCGCCCG
[153919] TTTACGCCG
[154660] ACGAATCCG
[156455] TCGATAGCG
[156569] CGCGTAGCG
[156617] CGATTAGCG
[156723] TATAACGCG
[156873] CGATACGCG
[156892] ATCTACGCG
[156915] TATTACGCG
[156921] CGTTACGCG
[157155] TAGTCCGCG
[157168] AATTCCGCG
[157234] GATAGCGCG
[157235] TATAGCGCG
[157236] ACTAGCGCG
[157244] ATTAGCGCG
[157283] TAGCGCGCG
[157296] AATCGCGCG
[157443] TAAATCGCG
[157518] GTACTCGCG
[157647] TTATTCGCG
[157897] CGATAGGCG
[159497] CGAATTGCG
[159527] TCGATTGCG
[159865] CGTCAATCG
[160313] CGTAGATCG
[160333] CTACGATCG
[160658] GACGTATCG
[161593] CGTATCTCG
[162061] CTAACGTCG
[162241] CAATCGTCG
[162377] CGACGGTCG
[162617] CGTATGTCG
[162761] CGATTGTCG
[163440] AATCGTTCG
[163577] CGTTGTTCG
[167060] ACCGATAGG
[167524] ACGCGTAGG
[168761] CGTATACGG
[169548] ATACGCCGG
[170212] ACGTAGCGG
[171155] TACGATCGG
[180839] TCGCGAATG
[190009] CGTAGCGTG
[190067] TATCGCGTG
[198969] CGTACGAAT
[200089] CGCGCTAAT
[200292] ACGCGTAAT
[200295] TCGCGTAAT
[200313] CGTCGTAAT
[206055] TCGTACGAT
[206227] TACGCCGAT
[206275] TAATCCGAT
[206436] ACGCGCGAT
[206461] CTTCGCGAT
[206495] TTCGGCGAT
[206759] TCGGTCGAT
[207161] CGTACGGAT
[209511] TCGCGATAT
[211431] TCGTCGTAT
[232601] CGCGATAGT
[233063] TCGCGTAGT
[233700] ACGTAACGT
[233875] TACGCACGT
[234087] TCGCGACGT

[234100] ACTCGACGT
[234392] AGCGTACGT
[234393] CGCGTACGT
[234724] ACGTACCGT
[235750] GCGTAGCGT
[235751] TCGTAGCGT
[235834] GGTACGCGT
[235916] ATAGCGCGT
[235923] TACGCGCGT
[236003] TAGTCGCGT
[236019] TATTCGCGT
[236156] ATTCGGCGT
[237479] TCGGTTCGT
[238793] CGATACGGT
[238905] CGTACCGGT
[239215] TTGCGCGGT
[246169] CGCGCAATT
[248297] CGGTCGATT
[249446] GCGCGTATT
[255206] GCGTACGTT
[255207] TCGTACGTT
[255782] GCGATCGTT

Number of missing fragments: 197

Supplemental Information S11: List of all human pathogenic viruses, a total of 199

Adeno-associated virus	Dependovirus,Parvoviridae	Human, vertebrates	Respiratory	None	NC_001401
Aichi virus	Kobuvirus,Picornaviridae	Human	Fecal-oral	Gastroenteritis	NC_001918
Australian bat lyssavirus	Lyssavirus,Rhabdoviridae	Human, bats	Zoonosis, animal bite	Fatal encephalitis	NC_003243
BK polyomavirus	Polyomavirus,Polyomaviridae	Human	Respiratory fluids or urine	None	NC_001538
Banna virus	Seadornavirus, Reoviridae	Human, cattle, pig, mosquitoes	Zoonosis, arthropod bite	Encephalitis	NC_004211, NC_004217, NC_004218, NC_004219, NC_004220, NC_004221, NC_004204, NC_004203, NC_004202, NC_004201, NC_004200
Barmah forest virus	Alphavirus,Togaviridae	Human, marsupials, mosquitoes	Zoonosis, arthropod bite	Fever, joint pain	NC_001786
Bunyamwera virus	Orthobunyavirus,Bunyaviridae	Human, mosquitoes	Zoonosis, arthropod bite	Encephalitis	NC_001925, NC_001926, NC_001927
Bunyavirus La Crosse	Orthobunyavirus,Bunyaviridae	Human, deer, mosquitoes, tamias	Zoonosis, arthropod bite	Encephalitis	NC_004108, NC_004109, NC_004110
Bunyavirus snowshoe hare	Orthobunyavirus,Bunyaviridae	Human, rodents, mosquitoes	Zoonosis, arthropod bite	Encephalitis	Not available
Cercopithecine herpesvirus	Lymphocryptovirus,Herpesviridae	Human, monkeys	Zoonosis, animal bite	Encephalitis	NC_006560
Chandipura virus	Vesiculovirus, Rhabdoviridae	Human, sandflies	Zoonosis, athropod bite	Encephalitis	Not available
Chikungunya virus	Alphavirus,Togaviridae	Human, monkeys, mosquitoes	Zoonosis, arthropod bite	Fever, joint pain	NC_004162
Cosavirus A	Cosavirus,Picornaviridae	Human	Fecal-oral (probable)	-	NC_012800
Cowpox virus	Orthopoxvirus, Poxviridae	Human, mammals	Zoonosis, contact	None	NC_003663

Coxsackievirus	Enterovirus,Picornaviridae	Human	Fecal-oral	Meningitis, myocarditis, paralysis	NC_001612
Crimean-Congo hemorrhagic fever virus	Nairovirus,Bunyaviridae	Human, vertebrates, ticks	Zoonosis, arthropod bite	Hemorrhagic fever	NC_005301, NC_005300, NC_005302
Dengue virus	Flavivirus,Flaviviridae	Human, mosquitoes	Zoonosis, arthropod bite	Hemorrhagic fever	NC_001477
Dhori virus	Thogotovirus,Orthomyxoviridae	Human, ticks	Zoonosis, arthropod bite	Fever, encephalitis	Not available
Dugbe virus	Nairovirus,Bunyaviridae	Human, ticks	Zoonosis, arthropod bite	Thrombocytopaenia	NC_004159, NC_004158, NC_004157
Duvenhage virus	Lyssavirus,Rhabdoviridae	Human, mammals	Zoonosis, animal bite	Fatal encephalitis	Not available
Eastern equine encephalitis virus	Alphavirus,Togaviridae	Human, birds, mosquitoes	Zoonosis, arthropod bite	Encephalitis	NC_003899
Ebolavirus	Ebolavirus,Filoviridae	Human, monkeys, bats	Zoonosis, contact	Hemorrhagic fever	NC_002549
Echovirus	Enterovirus,Picornaviridae	Human	Fecal-oral	Common cold	NC_001897
Encephalomyocarditis virus	Cardiovirus,Picornaviridae	Human, mouse, rat, pig	Zoonosis	Encephalitis	NC_001479
Epstein-Barr virus	Lymphocryptovirus,Herpesviridae	Human	Contact, saliva	Mononucleosis	NC_007605
European bat lyssavirus	Lyssavirus,Rhabdovirus	Human, bats	Zoonosis, animal bite	Fatal encephalitis	NC_009527
GB virus C/Hepatitis G virus	Pegivirus,Flaviviridae	Human	Blood, occasionally sexual	None	NC_001710
Hantaan virus	Hantavirus,Bunyaviridae	Human, rodents	Zoonosis, urine, saliva	Renal or respiratory syndrome	NC_005222, NC_005219, NC_005218
Hendra virus	Henipavirus,paramyxoviridae	Human, horse, bats	Zoonosis, animal bite	Encephalitis	NC_001906
Hepatitis A virus	Hepatovirus,picornaviridae	Human	Fecal-oral	Hepatitis	NC_001489
Hepatitis B virus	Orthohepadnavirus,Hepadnaviridae	Human, Chimpanzees	Sexual contact, blood	Hepatitis	NC_003977

Hepatitis C virus	Hepacivirus,Flaviviridae	Human	Sexual, blood	Hepatitis	NC_004102
Hepatitis E virus	Hepevirus,Unassigned	Human, pig, monkeys, some rodents, chicken	Zoonosis, food	Hepatitis	NC_001434
Hepatitis delta virus	Deltavirus,Unassigned	Human	Sexual contact, blood	Hepatitis	NC_001653
Horsepox virus	Orthopoxvirus, Poxviridae	Human, horses	Zoonosis, contact	None	Not available
Human adenovirus	Mastadenovirus,Adenoviridae	Human	Respiratory, fecal-oral	Respiratory	NC_001405
Human astrovirus	Mamastrovirus, Astroviridae	Human	Fecal-oral	Gastroenteritis	NC_001943
Human coronavirus	Alphacoronavirus,Coronaviridae	Human	Respiratory	Respiratory	NC_002645
Human cytomegalovirus	Cytomegalovirus,Herpesviridae	Human	Contact, urine, saliva	Mononucleosis, pneumonia	NC_001347
Human enterovirus 68, 70	Enterovirus,Picornaviridae	Human	Fecal-oral	Diarrhea, neurological disorder	NC_001430
Human herpesvirus 1	Simplexvirus,Herpesviridae	Human	Sexual contact, saliva	Skin lesions	NC_001806
Human herpesvirus 2	Simplexvirus,Herpesviridae	Human	Sexual contact, saliva	Skin lesions	NC_001798
Human herpesvirus 6A	Roseolovirus,Herpesviridae	Human	Respiratory, contact	Skin lesions	NC_001664
Human herpesvirus 6B	Roseolovirus,Herpesviridae	Human	Respiratory, contact	Skin lesions	NC_000898
Human herpesvirus 7	Roseolovirus,Herpesviridae	Human	Respiratory, contact	Skin lesions	NC_001716
Human herpesvirus 8	Rhadinovirus,Herpesviridae	Human	Sexual contact, saliva	Skin lymphoma	NC_009333
Human immunodeficiency virus	Lentivirus,Retroviridae	Human	Sexual contact, blood	AIDS	NC_001802
Human papillomavirus 1	Mupapillomavirus,Papillomaviridae	Human	Contact	Skin warts	NC_001356

Human papillomavirus 2	Alphapapillomavirus,Papillomaviridae	Human	Contact	Skin warts	NC_001352
Human papillomavirus 16,18	Alphapapillomavirus,Papillomaviridae	Human	Sexual	Genital warts,	NC_001526
Human parainfluenza	Respirovirus,Paramyxoviridae	Human	Respiratory	Respiratory	NC_003461
Human parvovirus B19	Erythrovirus,Parvoviridae	Human	Respiratory	Skin lesion	NC_000883
Human respiratory syncytial virus	Pneumovirus,Paramyxoviridae	Human	Respiratory	Respiratory	NC_001781
Human rhinovirus	Enterovirus,Picornaviridae	Human	Respiratory	Respiratory	NC_001617
Human SARS coronavirus	Betacoronavirus,Coronaviridae	Human, palm civet	Zoonosis	Respiratory	NC_004718
Human spumaretrovirus	Spumavirus,Retroviridae	Human	Contact, saliva	None	Not available
Human T-lymphotropic virus	Deltaretrovirus,Retroviridae	Human	Sexual contact, maternal-neonatal	Leukemia	NC_001436
Human torovirus	Torovirus,Coronaviridae	Human	Fecal-oral	Gastroenteritis	Not available
Influenza A virus	Influenzavirus A,Orthomyxoviridae	Human, birds, pigs	Respiratory or Zoonosis, animal contact	Flu	NC_002023, NC_002021, NC_002022, NC_002017, NC_002019, NC_002018, NC_002016 ,NC_002020
Influenza B virus	Influenzavirus B,Orthomyxoviridae	Human	Respiratory	Flu	NC_002204, NC_002205, NC_002206, NC_002207, NC_002208, NC_002209, NC_002210, NC_002211
Influenza C virus	Influenzavirus C,Orthomyxoviridae	Human	Respiratory	Flu	NC_006307, NC_006308, NC_006309, NC_006310, NC_006311, NC_006312, NC_006306

Isfahan virus	Vesiculovirus, Rhabdoviridae	Human, sandflies, gerbils	Zoonosis, arthropod bite	Undocumented, encephalitis?	Not available
JC polyomavirus	Polyomavirus,Polyomaviridae	Human	Fecal-oral or urine	Encephalitis	NC_001699
Japanese encephalitis virus	Flavivirus,Flaviviridae	Human, horses, birds, mosquitoes	Zoonosis, arthropod borne	Encephalitis	NC_001437
Junin arenavirus	Arenavirus,Arenaviridae	Human, rodents	Zoonosis, fomite	Hemorrhagic fever	NC_005081, NC_005080
KI Polyomavirus	Polyomavirus,Polyomaviridae	Human	Fecal-oral or urine	Encephalitis	NC_009238
Kunjin virus	Flavivirus,Flaviviridae	Human, horses, birds, mosquitoes	Zoonosis, arthropod borne	Encephalitis	Not available
Lagos bat virus	Lyssavirus,Rhabdoviridae	Human, mammals	Zoonosis, animal bite	Fatal encephalitis	Not available
Lake Victoria marburgvirus	Marburgvirus,Filoviridae	Human, monkeys, bats	Zoonosis, fomite	Hemorrhagic fever	NC_001608
Langat virus	Flavivirus,Flaviviridae	Human, ticks	Zoonosis, arthropod borne	Encephalitis	NC_003690
Lassa virus	Arenavirus,Arenaviridae	Human, rats	Zoonosis, fomites	Hemorrhagic fever	NC_004297, NC_004296
Lordsdale virus	Norovirus,Caliciviridae	Human	Fecal-oral	Gastroenteritis	Not available
Louping ill virus	Flavivirus,Flaviviridae	Human, mammals, ticks	Zoonosis, arthropod bite	Encephalitis	NC_001809
Lymphocytic choriomeningitis virus	Arenavirus,Arenaviridae	Human, rodents	Zoonosis, fomite	Encephalitis	NC_004291, NC_004294
Machupo virus	Arenavirus,Arenaviridae	Human, monkeys, mouse	Zoonosis, fomite	Encephalitis	NC_005079, NC_005078
Mayaro virus	Alphavirus,Togaviridae	Human, mosquitoes	Zoonosis, arthropod bite	Fever, joint pain	NC_003417
MERS coronavirus	Betacoronavirus,Coronaviridae	Human, Tomb bat	Zoonosis	Respiratory	NC_019843
Measles virus	Morbilivirus,Paramyxoviridae	Human	Respiratory	Fever, rash	NC_001498

Mengo encephalomyocarditis virus	Cardiovirus,Picornaviridae	Human, mouse, rabbit	Zoonosis	Encephalitis	Not available
Merkel cell polyomavirus	Polyomavirus,Polyomaviridae	Human	-	Merkel cell carcinoma	NC_010277
Mokola virus	Lyssavirus,Rhabdoviridae	Human, rodents, cat, dog shrew	Zoonosis, animal bite	Encephalitis	NC_006429
Molluscum contagiosum virus	Molluscipoxvirus,Poxviridae	Human	Contact	Skin lesions	NC_001731
Monkeypox virus	Orthopoxvirus, Poxviridae	Human, mouse, prairie dog	Zoonosis, contact	Skin lesions	NC_003310
Mumps virus	Rubulavirus,Paramyxoviridae	Human	Respiratory, saliva	Mumps	NC_002200
Murray valley encephalitis virus	Flavivirus,Flaviviridae	Human, mosquitoes	Zoonosis, arthropod bite	Encephalitis	NC_000943
New York virus	Hantavirus,Bunyavirus	Human, mouse	Zoonosis, urine, saliva	Hemorrhagic fever	Not available
Nipah virus	Henipavirus,Paramyxoviridae	Human, bats	Zoonosis, animal bite	Encephalitis	NC_002728
Norwalk virus	Norovirus,Caliciviridae	Human	Fecal-oral	Gastroenteritis	NC_001959
O'nyong-nyong virus	Alphavirus,Togaviridae	Human, mosquitoes	Zoonosis, arthropod bite	Fever, joint pain	NC_001512
Orf virus	Parapoxvirus,Poxviridae	Human, mammals	Zoonosis, contact	Skin lesions	NC_005336
Oropouche virus	Orthobunyavirus,Bunyaviridae	Human, wild animals(sloths)	Zoonosis, arthropod bite	Fever, joint pain	NC_005777, NC_005775, NC_005776
Pichinde virus	Arenavirus,Arenaviridae	Human, rat, guinea pig	Zoonosis, fomite	Hemorrhagic fever	NC_006447, NC_006439
Poliovirus	Enterovirus,Picornaviridae	Human, mammals	Fecal-oral	Poliomyelitis	NC_002058
Punta toro phlebovirus	Phlebovirus,Bunyaviridae	Human, sandflies	Zoonosis, arthropod bite	Hemorrhagic fever	Not available
Puumala virus	Hantavirus,Bunyavirus	Human, bank vole	Zoonosis, urine, saliva	Hemorrhagic fever	NC_005223, NC_005224, NC_005225

Rabies virus	Lyssavirus,Rhabdoviridae	Human, mammals	Zoonosis, animal bite	Fatal encephalitis	NC_001542
Rift valley fever virus	Phlebovirus,Bunyaviridae	Human, mammals, mosquitoes, sandflies	Zoonosis, arthropod bite	Hemorrhagic fever	NC_002043, NC_002044, NC_002045
Rosavirus A	Rosavirus,Picornaviridae	Human			NC_024070
Ross river virus	Alphavirus,Togaviridae	Human, mosquitoes, marsupials	Zoonosis, arthropod bite	Fever, joint pain	NC_001544
Rotavirus A	Rotavirus,Reoviridae	Human	Fecal-oral	Gastroenteritis	NC_011507, NC_011506, NC_011508, NC_011510, NC_011500, NC_011509, NC_011501, NC_011502, NC_011503, NC_011504, "NC_011505,
Rotavirus B	Rotavirus,Reoviridae	Human	Fecal-oral	Gastroenteritis	NC_007548, NC_007549, NC_007550, NC_007551, NC_007552, NC_007553, NC_007554, NC_007555, NC_007556, NC_007557, NC_007558
Rotavirus C	Rotavirus,Reoviridae	Human	Fecal-oral	Gastroenteritis	NC_007547, NC_007546, NC_007572, NC_007574, NC_007570, NC_007543, NC_007544, NC_007571, NC_007545, NC_007569, NC_007573
Rubella virus	Rubivirus,Togaviridae	Human	Respiratory	Rubella	NC_001545

Sagiyama virus	Alphavirus,Togaviridae	Human, horse, pig, mosquitoes	Zoonosis, arthropod bite	Fever, joint pain	Not available
Salivirus A	Salivirus,Picornaviridae	Human		Gastroenteritis	NC_012957
Sandfly fever sicilian virus	Phlebovirus,Bunyaviridae	Human, sandflies	Zoonosis, arthropod bite	Hemorrhagic fever	Not available
Sapporo virus	Sapovirus,Caliciviridae	Human	Fecal-oral	Gastroenteritis	NC_006554
Semliki forest virus	Alphavirus,Togaviridae	Human, birds, hedgehog, mosquitoes	Zoonosis, arthropod bite	Fever, joint pain	NC_003215
Seoul virus	Hantavirus,Bunyavirus	Human, rats	Zoonosis, urine, saliva	Hemorrhagic fever	NC_005236, NC_005237, NC_005238
Simian foamy virus	Spumavirus,Retroviridae	Human, monkeys	Zoonosis, contact	None	NC_001364
Simian virus 5	Rubulavirus,Paramyxoviridae	Human, dog	Zoonosis, contact	Undocumented	Not available
Sindbis virus	Alphavirus,Togaviridae	Human, birds, mosquitoes	Zoonosis, arthropod bite	Fever, joint pain	NC_001547
Southampton virus	Norovirus,Caliciviridae	Human	Fecal-oral	Gastroenteritis	Not available
St. louis encephalitis virus	Flavivirus,Flaviviridae	Human, birds, mosquitoes	Zoonosis, arthropod bite	Encephalitis	NC_007580
Tick-borne powassan virus	Flavivirus,Flaviviridae	Human, ticks	Zoonosis, arthropod bite	Encephalitis	NC_003687
Torque teno virus	Alphatorquevirus,Anelloviridae	Human	Sexual, blood	None	NC_002076
Toscana virus	Phlebovirus,Bunyaviridae	Human, mosquitoes	Zoonosis, arthropod bite	Hemorrhagic fever	NC_006318, NC_006319, NC_006320
Uukuniemi virus	Phlebovirus,Bunyaviridae	Human, ticks	Zoonosis, arthropod bite	Hemorrhagic fever	NC_005214, NC_005220, NC_005221
Vaccinia virus	Orthopoxvirus, Poxviridae	Human, mammals	Contact	None	NC_006998
Varicella-zoster virus	Varicellovirus,Herpesviridae	Human	Respiratory, contact	Varicella	NC_001348
Variola virus	Orthopoxvirus, Poxviridae	Human	Respiratory	Variola	NC_001611

Venezuelan equine encephalitis virus	Alphavirus,Togaviridae	Human, rodents, mosquitoes	Zoonosis, arthropod bite	Fever, joint pain	NC_001449
Vesicular stomatitis virus	Vesiculovirus, Rhabdoviridae	Human, cattle, horse, pig, flies	Zoonosis, athropod bite	Encephalitis	NC_001560
Western equine encephalitis virus	Alphavirus,Togaviridae	Human, vertebrates, mosquitoes	Zoonosis, arthropod bite	Fever, joint pain	NC_003908
WU polyomavirus	Polyomavirus,Polyomaviridae	Human	Respiratory fluids or urine	None	NC_009539
West Nile virus	Flavivirus,Flaviviridae	Human, birds, ticks, mosquitoes	Zoonosis, arthropod bite	Hemorrhagic fever	NC_001563
Yaba monkey tumor virus	Orthopoxvirus, Poxviridae	Human, monkeys	Zoonosis, contact	None	NC_005179
Yaba-like disease virus	Orthopoxvirus, Poxviridae	Human, monkeys	Zoonosis, contact	None	NC_002642
Yellow fever virus	Flavivirus,Flaviviridae	Human, monkeys, mosquitoes	Zoonosis, arthropod bite	Hemorrhagic fever	NC_002031
Zika virus	Flavivirus,Flaviviridae	Human, monkeys, mosquitoes	Zoonosis, arthropod bite	Fever, joint pain, rash	NC_012532
